# Gel Polymer Electrolytes: Advancing Solid-State Batteries for High-Performance Applications

**DOI:** 10.3390/gels9070585

**Published:** 2023-07-21

**Authors:** Kanakaraj Aruchamy, Subramaniyan Ramasundaram, Sivasubramani Divya, Murugesan Chandran, Kyusik Yun, Tae Hwan Oh

**Affiliations:** 1 School of Chemical Engineering, Yeungnam University, Gyeongsan 38541, Republic of Korea; a.kanakaraj@yu.ac.kr (K.A.); ramasundaram79@hotmail.com (S.R.); divi.fysics@gmail.com (S.D.); 2 Department of Bionanotechnology, Gachon University, Seongnam-si 13120, Republic of Korea; chandranchem534@gmail.com (M.C.); ykyusik@gachon.ac.kr (K.Y.)

**Keywords:** gel polymer electrolytes, solid-state batteries, electric vehicles, renewable energy, energy storage solutions

## Abstract

Gel polymer electrolytes (GPEs) hold tremendous potential for advancing high-energy-density and safe rechargeable solid-state batteries, making them a transformative technology for advancing electric vehicles. GPEs offer high ionic conductivity and mechanical stability, enabling their use in quasi-solid-state batteries that combine solid-state interfaces with liquid-like behavior. Various GPEs based on different materials, including flame-retardant GPEs, dendrite-free polymer gel electrolytes, hybrid solid-state batteries, and 3D printable GPEs, have been developed. Significant efforts have also been directed toward improving the interface between GPEs and electrodes. The integration of gel-based electrolytes into solid-state electrochemical devices has the potential to revolutionize energy storage solutions by offering improved efficiency and reliability. These advancements find applications across diverse industries, particularly in electric vehicles and renewable energy. This review comprehensively discusses the potential of GPEs as solid-state electrolytes for diverse battery systems, such as lithium-ion batteries (LiBs), lithium metal batteries (LMBs), lithium–oxygen batteries, lithium–sulfur batteries, zinc-based batteries, sodium–ion batteries, and dual-ion batteries. This review highlights the materials being explored for GPE development, including polymers, inorganic compounds, and ionic liquids. Furthermore, it underscores the transformative impact of GPEs on solid-state batteries and their role in enhancing the performance and safety of energy storage devices.

## 1. Introduction

The electrolyte plays a crucial role in batteries as it separates the anode and cathode terminals. It enables the reversible transport of ions between these terminals, enabling the conversion of stored chemical energy into electrical energy [1,2,3]. The practical applications of electrolytes in batteries depend on important properties including electrochemical stability, ionic conductivity, and thermal stability [4,5]. Traditionally, organic liquid electrolytes have been widely used in lithium-ion batteries (LIBs) [6]. However, the use of organic liquid electrolytes presents significant operational safety concerns, including leakage, flammability, combustion, and electrochemical instability [7,8]. These risks make organic liquid electrolytes a potentially hazardous option for applications such as electric vehicles [9,10]. Moreover, the leakage of a liquid electrolyte during prolonged charging/discharging cycles can result in the loss of active battery materials. Therefore, there is a growing need for sustainable and safer electrolyte technologies that can provide high levels of safety [11,12]. Ceramic and solid polymer electrolytes have been investigated as promising alternatives to liquid electrolytes [13,14]. Polymer electrolytes offer several advantages, including noncombustible properties, no risk of internal short circuits, and no leakage issues. However, solid electrolytes face challenges such as low ionic conductivity and weak electrolyte–electrode interfaces [15]. In the past, solid polymer electrolytes based on poly(ethylene oxide) (PEO) and alkaline metal salts were developed, but they exhibited poor ionic conductivity (10^−8^ S cm^−1^) and limited cyclic performance (200 to 300 cycles), resulting in overall poor battery performance [16,17]. Additionally, solid polymer electrolytes have high crystallinity, which reduces the ionic conductivity at ambient temperatures. The solid nature of these electrolytes also leads to poor electrode interface formation, resulting in insufficient contact and larger polarization effects during charge/discharge cycles [18,19].

In terms of establishing a stronger interface with electrodes, gel polymer electrolytes (GPEs) offer advantages over solid electrolytes. Unlike liquid or solid electrolytes, GPEs possess a unique combination of properties, exhibiting both the diffusive characteristics of liquids and the cohesive properties of solids [20,21]. GPEs consist of a solid polymer matrix that encapsulates or stores liquid electrolytes. This design allows GPEs to serve as both the electrolyte and separator, eliminating the risk of electrolyte leakage [22]. The incorporation of electrolytes within the gel matrix significantly enhances the ionic conductivity at ambient temperatures and improves the stability of the electrolyte–electrode interface. The ionic conductivity of GPEs at ambient temperature is estimated to range from 10^−4^ to 10^−3^ S/cm, which is comparable to commercial liquid electrolytes. This high ionic conductivity, combined with the operational safety, mechanical flexibility, and non-combustible nature of GPEs, makes them suitable for use in various battery types, including lithium-ion batteries, sodium-ion batteries, zinc–air batteries, and aluminum–air batteries [23,24,25]. The formation of a GPE involves blending the electrolyte with high-ionic-conductivity materials such as potassium hydroxide, lithium salts, or ionic liquids, which are mixed with an appropriate polymer matrix [26,27]. This mixture allows for the formation of a gel. GPEs can be fabricated as films or coatings, offering versatility in their application [28]. 

The polymers used as matrixes for gel polymer electrolytes (GPEs) need to meet specific requirements. The liquid electrolytes are formed by dissolving salts in solvents. Common salt electrolytes include bis-(trifluoromethane)sulfonimide lithium (LiTFSI), LiClO_4_, LiSnOS, and sodium bis(fluorosulfonyl)imide [29]. To incorporate a salt into a polymer matrix via composite-type solution mixing, the polymer matrix should be soluble in polar solvents. Therefore, the polymers chosen for GPEs must possess polar functional groups [30]. Additionally, these polar functional groups can interact with the liquid electrolyte, thereby improving ion transport. When preparing GPEs via impregnating the polymer matrix with a liquid electrolyte, the amorphous portion of the polymer accommodates the liquid electrolytes and facilitates ion transfer. Consequently, polymers with low glass transition temperatures and low levels of crystallinity are preferred [31]. These characteristics allow the polymer to effectively host the liquid electrolyte, enabling ion movement. Moreover, polymers with high mechanical, thermal, and chemical stabilities are desirable for GPEs as they contribute to achieving a wide electrochemical window [32,33].

Commonly used polymer matrixes for gel polymer electrolytes (GPEs) include poly(vinylidene fluoride) (PVDF), poly(vinylidene fluoride-hexafluoropropylene) (PVDF-HFP), poly(methyl methacrylate) (PMMA), polyacrylonitrile (PAN), and polyethylene oxide (PEO) [25,31]. PVDF and PVDF-HFP are potential choices for GPE matrixes due to their high dielectric constants and the presence of strong electron-withdrawing C–F bonds [34,35]. In particular, PVDF-HFP is known for its high chemical resistance and mechanical strength. The incorporation of hexafluoropropylene (HFP) units into the PVDF matrix reduces the overall crystallinity and lowers the melting point of PVDF-HFP. This lower crystallinity enhances the swelling of PVDF-HFP by solvents, even allowing it to be solubilized by acetone. The high swelling capability of PVDF-HFP enables the easy uptake of liquid electrolytes into GPE matrixes based on this polymer. PEO-based GPEs are also considered promising [36]. PEO is cost-effective and can form GPE films with reasonable mechanical and thermal stabilities. Additionally, the CH_2_CH_2_O repeating unit in the PEO backbone exhibits a strong interaction with Li ions, enabling better charge transport [37,38]. However, there are challenges that need to be addressed. The chemical environment of a lithium-ion battery (LIB) is complex and often leads to the dissolution of lithium polysulfides in the electrolyte. To prevent the growth of lithium dendrites, a mechanically stable gelling matrix is required.

In order to meet the practical application requirements of batteries, gel polymer electrolytes (GPEs) with excellent affinity to electrodes, high ionic conductivity, and cyclic stability have been developed. However, there is often a trade-off between mechanical strength and ionic stability when attempting to improve these properties. Therefore, various combinations of GPEs have been prepared with the objective of achieving the following merits: good mechanical and thermal stabilities, a high level of ionic conductivity comparable to that of liquid electrolytes at an ambient temperature, a high transference number, and a favorable electrolyte and electrode interface. 

Significant progress has been made in developing GPEs with improved properties, leading to their widespread application in various battery systems. This comprehensive review aims to explore the advancements in GPE technology which have greatly transformed energy storage solutions, particularly with respect to batteries with high energy densities and safety. Within this review, we extensively discuss different types of GPEs, including GPEs with flame-retardant properties, GPEs that prevent dendrite formation, hybrid solid-state batteries, and GPEs that are suitable for 3D printing. Moreover, we investigate the methods used to prepare GPEs, such as solution casting, phase inversion, in situ polymerization, and electrospinning. We provide detailed coverage of a wide range of battery systems, including lithium-ion batteries (LiBs), lithium metal batteries (LMBs), lithium–oxygen batteries, lithium–sulfur batteries, zinc-based batteries, sodium-ion batteries, and dual-ion batteries. Additionally, we highlight the materials currently being studied for GPE development, including polymers, inorganic compounds, and ionic liquids. By presenting the latest advancements and their impacts on solid-state batteries, this review aims to provide a comprehensive understanding of the potential of GPEs in improving the performance and safety of energy storage devices. The insights presented in this review are highly valuable to researchers, industry professionals, and enthusiasts involved in advanced battery technologies.

## 2. Methods for the Preparation of Gel Polymer Electrolytes

**Figure 1:**
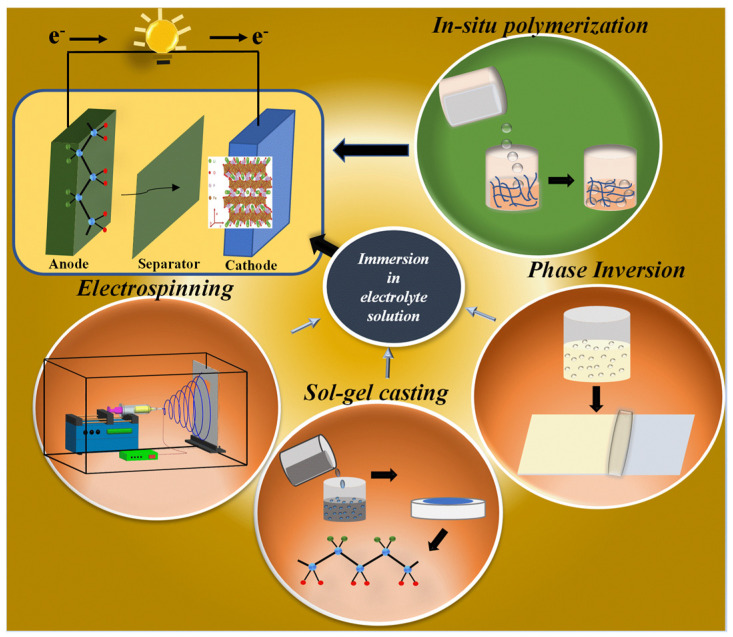
Schematic representation of various preparation methods for gel polymer electrolytes.

In a GPE, the polymer chains form a network with interconnected pores that facilitate the absorption of the liquid electrolyte and enable charge transfer. GPEs can be prepared using two different methods, depending on the nature of the preparation process. One method involves using a porous polymer matrix that allows the liquid electrolyte to be absorbed through swelling. Porous structures in the polymer matrix can be achieved through the controlled removal of a solvent from a polymer solution or by directly forming fibrous structures with inherent porosity. The other method involves storing the liquid electrolyte between polymer chains generated via in situ polymerization. The common methods used for preparing GPEs include solution casting, phase inversion, and in situ polymerization. These methods are considered energy-efficient, cost-effective, and environmentally friendly compared to other available methods for GPE preparation [39,40,41]. A schematic representation of various preparation methods for gel polymer electrolytes is shown in Figure 1.

### 2.1. Solution Casting

Solution casting is considered advantageous compared to other methods due to its simplicity and the absence of specialized equipment requirements. In this technique, the matrix polymer and salt electrolytes are dissolved in a solvent, forming a homogeneous solution. The solution is then spread onto a suitable substrate and allowed to dry at a low temperature, typically below 60 °C. Solvents with low boiling points are usually selected to facilitate the easy removal of the solvent at low temperatures. The low processing temperature restricts the movement of polymer chains, resulting in a less crystalline polymer matrix. Solution-cast GPEs offer several benefits. They are easy to process, flexible, compact, and allow for the tuning of their weight and thickness. They exhibit resistance to pressure and are non-volatile. However, due to their low crystallinity and the presence of salt, solution-cast GPEs may have lower levels of mechanical strength. To address this, crosslinking agents can be added to connect the matrix polymer chains, thereby enhancing the mechanical properties. It is important to note that electrolytes, especially lithium salts, are sensitive to moisture. Therefore, strict measures must be taken to avoid any interference from moisture or water during the solution casting process.

In the case of PVDF and PVDF-HFP, the use of high-boiling-point solvents such as *N,N*′-dimethylformamide, *N,N*′-dimethylacetamide, and N-methylpyrrolidone is unavoidable. While these solvents can be blended with up to 30% acetone to aid in dissolving these polymers, temperatures above 50 °C are required to effectively remove these solvents and obtain GPEs with sufficient mechanical strength [37,39,42]. Therefore, to achieve a GPE with an adequate amorphous phase, plasticizers such as dibutyl phthalate, carbonate solvents (ethylene carbonate and propylene carbonate), and polyethylene glycol are commonly used. In some cases, water-forming agents are incorporated into the polymer matrix, and they are subsequently removed by treating the GPE matrix with a suitable solvent, similar to the extraction process used for plasticizers. Once the plasticizers and pore-forming agents are removed, the GPE matrix is activated by immersing it in a liquid electrolyte solution. The pores created in the GPE matrix and the void spaces left by the removal of plasticizers and pore-forming agents are filled with the liquid electrolytes. The quantity of liquid electrolytes trapped in the GPE matrix directly impacts the overall ionic conductivity, with higher quantities leading to increases in conductivity. By carefully selecting the appropriate solvents, plasticizers, and pore-forming agents, GPEs with desired properties can be obtained. The removal of solvents and additives must be performed effectively to ensure the mechanical strength and stability of the resulting GPE. The incorporation of liquid electrolytes into the activated GPE matrix is crucial for enhancing the ionic conductivity and overall performance of the GPE.

### 2.2. Phase Inversion Method

The phase inversion method is a relatively simple and cost-effective technique that holds great potential for scalability. In this method, a polymer solution or a polymer–liquid composite is solidified through the introduction of a non-solvent. The process involves coating the polymer solution onto a substrate, such as glass or between two glass slides, and immersing this system in a non-solvent. During the phase inversion process, the non-solvent replaces the solvent in the polymer solution, leading to the precipitation and solidification of the polymer. The addition of oligomers as additives can enhance the porosity of the resulting GPE by being extracted out during phase inversion. By carefully tuning the experimental conditions and the composition of the polymer solution, GPEs with honeycomb-like and network morphologies can be achieved. The formation of a network-like morphology with a narrow pore distribution helps to prevent the leakage of liquid electrolytes and enables the GPE to exhibit high ionic conductivity. The phase inversion method offers advantages such as the production of continuous and defect-free GPEs [43,44,45]. However, it requires the meticulous optimization of the solvent, non-solvent, and the concentration of the polymer solution to achieve desirable results. It is important to note that this method can lead to the organic contamination of water, raising significant environmental concerns.

### 2.3. In Situ Polymerization

In the in situ polymerization method, polymerization or the growth of polymer chains occurs in the presence of a liquid electrolyte. The monomer or prepolymer, initiators, and the liquid electrolyte to be loaded in the gel polymer electrolyte (GPE) are mixed together in a matrix and subjected to polymerization under light or heat. This process enables the formation of GPEs with uninterrupted ion transport networks [40,41]. When the premixture is coated on the electrode and subjected to polymerization, the contact resistance between the electrode and electrolyte can be reduced. This reduction in electrolyte–electrode interfacial resistance effectively hinders problems such as the growth of lithium dendrites. The advantages of this method include low viscosity, easy handling, and the formation of a good interface [46]. However, the materials required for in situ polymerization are expensive. Unlike bulk polymers such as PVDF, PEO, and PVA, the thermal stability of GPEs grown via in situ polymerization is highly limited.

### 2.4. Electrospinning

In the electrospinning method, the polymer matrix is transformed into a porous layer of nanofibers. A sufficient voltage is applied to a polymer solution with the required viscosity, which is then drawn into fibers through a conducting nozzle. These fibers are collected on a conducting substrate which serves as the bottom electrode. The thickness of the fibrous layer can be controlled by adjusting the volume of the polymer solution. Parameters such as the concentration of the polymer solution, the applied voltage, the inner diameter of the nozzle, and the boiling point of the solvent are optimized to control the thickness of the fibers. The electrospun polymeric layer is utilized as a matrix for the gel polymer electrolyte (GPE). The electrolyte is loaded by impregnating a liquid electrolyte solution with the fibrous matrix. The interconnected pores within the fibrous structures provide a larger surface area and efficient ion-conducting channels [47,48]. PVDF, PVDF-HFP, and PAN have been employed as electrospun matrixes for GPEs. Electrospinning offers several advantages, including reducing the use of solvents, preventing the loss of polymer and active materials, and the rapid solidification of the polymer solution. However, it requires a polymer solution with a high viscosity. In the case of PVDF, a minimum concentration ranging from 12 to 18 wt% is necessary to obtain an intact nanofiber layer. Additionally, caution must be exercised during solution preparation. The solvents for PVDF, such as *N,N*′-dimethylformamide (boiling point = 153 °C) and *N,N*′-dimethylacetamide (boiling point = 165 °C), are non-volatile. To facilitate solidification, up to 30% of acetone (boiling point = 56 °C) is added. It is important to note that concentrated polymer solutions are more prone to drying and should not be stored for extended periods [37,49,50].

## 3. Gel Polymer Electrolytes for Li-Ion Batteries

Lithium-ion batteries (LIBs) utilize the reversible reduction of lithium ions for energy storage. They have emerged as a promising type of energy storage device due to their high energy density, long lifespan, and low self-discharge properties. LIBs have gained a dominant position in the market and are considered ideal choices for powering portable electronic devices, electric vehicles, and implantable medical devices. However, the use of liquid electrolytes in LIBs raises intrinsic safety concerns, such as the potential for overheating, which can result in bursting and catastrophic thermal accidents. In 1975, PEO-based gel polymer electrolytes (GPEs) for LIBs were developed by Wright [51]. Since then, significant progress has been made in the development of GPEs specifically tailored for LIB applications. These advancements are discussed in detail in this section, focusing on the recent developments in GPE technology for lithium-ion batteries. Table 1 presents a comparison of diverse GPE compositions for Li-ion batteries, encompassing details such as method of preparation, specific capacity, cyclic stability, and ionic conductivity.

Li superionic conductor (LISICON)-type solid electrolytes are known to be promising, but they suffer from a hygroscopic nature and limited chemical stability. In an effort to address these challenges, Kuo et al. developed a LISICON-based gel polymer electrolyte (GPE) using a PVDF-HFP matrix with varying compositions of LiSnOS. Instead of the moisture-sensitive Li_2_S, LiSnOS was used as a precursor to avoid the hygroscopic nature. LiSnOS was prepared via the sulfurization of LiNO_3_ with SnOS. By adjusting the composition of LiSnOS, the highest Li ion conductivity achieved was 1.92 × 10^−4^ S cm^−1^. A GPE containing 30 wt% LiSnOS exhibited the highest discharge capacity of 134.6 mA h/g at 0.2 C and remained stable over 30 cycles. A GPE with 30% LiSnOS facilitated fast ion transport and proved to be beneficial for maintaining flexibility and flame retardancy in lithium-ion batteries (LiBs) [52].

Three-dimensional printing is a valuable technique for fabricating layered and complex structures. Gambe et al. developed a UV-curable gel polymer electrolyte (GPE) using 3D printing. This method allows for the direct printing of the GPE onto various substrates at room temperature, making it suitable for processing thermally unstable materials. The electrolyte ink was formulated using a UV-curable monomer, ionic gels, an ionic liquid (IL) capable of conducting lithium, and silica nanoparticles. In this study, lithium(trifluoromethanesulfonyl)imide (Li-TFSI) was dissolved in 1-ethyl-3-methyl imidazolium bis(trifluoromethanesulfonyl) imide (EMI-TFSI) and used as an Li-conducting IL. The structural stability of the GPE was optimized by varying the concentration of the silica nanoparticles. A mixture of bis(trifluoromethanesulfonyl) imide powder, 1-ethyl-3-methyl imidazolium bis(trifluoromethanesulfonyl)imide, 2-hydroxyethyl methacrylate (a monomer of poly(hydroxyethylmethacrylate)—P(HEMA)), silica nanoparticles, ethylene glycol dimethacrylate (a crosslinker), and 2,2-dimethoxy-2-phenylacetophenone as a photo initiator was prepared at an appropriate ratio to serve as a 3D printable ink. The printed patterns were then exposed to UV light to form the GPE. Quasi solid-state lithium-ion batteries (LiBs) were assembled by printing the GPE ink onto cathodes coated with a slurry of lithium cobalt oxide and a lithium foil anode. Fully 3D printed LiBs were obtained by using a 3D printed lithium cobalt oxide cathode and a lithium titanium oxide anode. This 3D printing approach enabled the simple fabrication of flexible LiBs. The initial capacity of the batteries was approximately 100 mA h/g (at 0.1 C). The ionic conductivity of the GPE, regardless of the filler content (ranging from 3 to 7 wt.%), was measured to be 2.9 × 10^−3^ S/cm at 25 °C. The ionic conductivity of five layers of 3D printed GPE (240 × 175 μm) was 2 × 10^−3^ S/cm. Good adhesion was observed in the 3D printed GPE, and there was no contact resistance. The number of interfaces in the 3D printed layers was considered to be larger than that in molded GPE samples [53]. 

Due to their electrical stability, high ionic conductivity, and mechanical strength, garnet Li_7_La_3_Zr_2_O_12_ (LLZO) materials with tantalum (Ta) doping (referred to as LLZTO) have emerged as potential candidates for solid-state electrolytes, despite the challenge of large interface resistances arising from inactive cathodes and the inherently difficult garnet–electrode interfaces. In a study by Meng Liu et al., a cathode material was prepared using a mass ratio of 5:5:90 of carbon black, poly(vinylidene fluoride) (PVDF), and LiCoO_2_. The cathode was fabricated by mixing the components into a slurry, coating it onto aluminum foil, and subsequently drying it. The anode material consisted of lithium metal. For the solid-state electrolyte, a precursor mixture for the garnet electrolyte was prepared using ethylene glycol diacrylate and coated onto an LLZO pellet. Thermally induced in situ polymerization was employed to form a poly(ethylene glycol) diacrylate (PEGDA) layer at 60 °C for 1 h. The incorporation of this layer led to a decrease in the interface resistance from 6880 to 473 Ω. Furthermore, a Li symmetric cell demonstrated a stable galvanostatic charge/discharge profile for 400 h without the formation of lithium dendrites. Additionally, a solid-state Li|GPE@LLZO|LiCoO_2_ battery exhibited a retention of the initial discharge capacity (104.1 mA h/g) of 82.6% after 100 cycles (at 0.5 C) at room temperature. These findings highlight the potential of garnet LLZO-based electrolytes to reduce interface barriers and improve compatibility in solid-state lithium-ion batteries [54].

**Figure 2:**
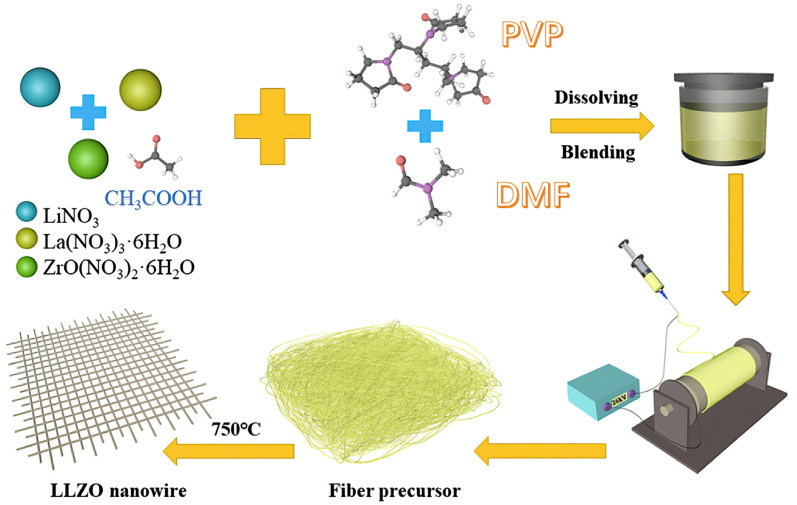
Li_7_La_3_Zr_2_O_12_ (LLZO) nano network experimental procedure. Reproduced with permission from [55].

To overcome the disadvantages associated with liquid electrolytes, such as electrolyte leakage and the risk of explosions due to lithium dendrite formation, solid-state electrolytes (SSEs) have gained significant attention in recent years. In a study by Haoshan Xu et al., cathode materials were prepared using 80 wt% LiFePO_4_, 10 wt% PEO/TPU/LiTFSI composite, and 10 wt% carbon black, with N-methyl pyrrolidone (NMP) serving as the solvent medium on an aluminum foil substrate. A polyethylene oxide (PEO)/thermoplastic polyurethane (TPU)/Li_7_La_3_Zr_2_O_12_ (LLZO) nano-network composite electrolyte was prepared using the solution casting method. LLZO nanonetworks were synthesized via electrospinning, followed by thermal treatment. A mixture of LiNO_3_, La(NO_3_)_3_⋅6H_2_O, and ZrO(NO_3_)_2_⋅6H_2_O in stoichiometric proportions was prepared. To compensate for lithium loss during calcination, more than 15 wt% of LiNO_3_ was added. Polyvinylpyrrolidone (PVP) was dissolved in N,N-dimethylformamide (DMF) at a concentration of 8 wt% and added to a solution containing glacial acetic acid mixed with deionized water. The PVP and salt solution were mixed at a weight ratio of 1:1.2 and stirred at 60 °C for 8 h. The resulting mixture was electrospun at a high voltage, with a distance of 18 cm between the needle and the nanowire collector, and a constant voltage of 26 kV was maintained. The electrospun precursor was pre-oxidized at 280 °C for 2 h, followed by calcination at 750 °C for 2 h in an air atmosphere with a heating rate of 1.0 °C/min to obtain the LLZO nanonetwork. The preparation process is illustrated in Figure 2. This composite electrolyte exhibited a maximum ionic conductivity of 1.33 × 10^−3^ S cm^−1^ at 60 °C and a high electrochemical stability window of 5.6 V. To enhance the electrochemical properties and mechanical stability, thermoplastic polyurethane (TPU) was widely employed. The discharge capacity was observed to be 170 mA h g^−1^ at 0.1 C after 100 cycles, demonstrating improved cycle durability. At 0.5 C and 60 °C, the initial capacity retention was 96.1%. Solid polymer composite electrolytes offer several advantages, including a high degree of safety and excellent electrochemical performance. As a result, solid-state cells hold great promise as future candidates for lithium batteries [55].

**Figure 3:**
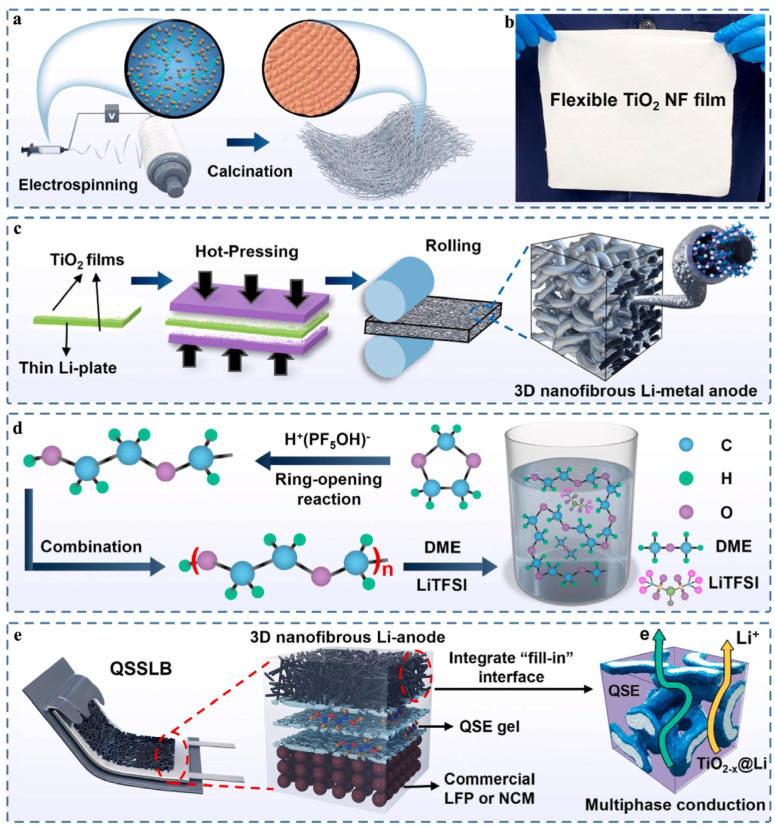
(**a**) The process of creating a flexible TiO_2_ nanofiber film involves sol–gel electrospinning, followed by calcination. This is accomplished by first creating a sol–gel solution and passing it through an electrospinning apparatus to produce nanofibers. These fibers are then calcined to form the final TiO_2_ film. (**b**) A flexible TiO_2_ nanofiber film, similar to the one created in the lab, can be produced through the above process. (**c**) A hot–rolling technique can be employed to produce a 3D nanofibrous TiO_2_–_x_@Li anode. This involves rolling a pre-prepared TiO_2_–_x_@Li material to produce a highly compacted and conductive anode. (**d**) Highly ionic conductive quasi-solid electrolytes (QSEs) can be created via the in situ polymerization of DOL at room temperature. This process involves the polymerization of DOL to produce a solid electrolyte material that is highly conductive. (**e**) A quasi–solid–state Li-battery (QSSLB) can be created by integrating multiple conduction paths. This battery contains multiphase conduction paths that allow for the efficient transfer of charge and can be used in a variety of applications. Reproduced with permission from [56].

The integration of nanofibrous TiO_2_-x@Li anodes and a highly conductive solid-state electrolyte (GPE) by Jiao et al. not only addresses the challenges associated with Li-metal anodes but also presents a promising solution for enhancing the performance and commercial viability of lithium-ion batteries (LIBs). Their initial findings demonstrate the effectiveness of utilizing nanofibrous TiO_2_-x@Li anodes and a highly conductive solid-state electrolyte (GPE) to enhance the stability of the Li-metal anode and improve its compatibility with electrolytes. The GPE was prepared by dissolving LiTFSI and LiPF_6_ in DMF with 1,3-dioxolane (DOL) and allowing it to form poly(dioxolane) via a 6 h room temperature reaction. The nanofibrous TiO_2_-x@Li anodes promote the uniform deposition of Li by facilitating the conduction of Li ions along the GPE and their deposition onto the electronically conductive TiO_2_-x nanofibers. When combined with high-loading cathodes such as LiFePO_4_ or LiNi_1/3_Co_1/3_Mn_1/3_O_2_, the quasi-solid-state Li batteries exhibited excellent long-term cycling stability (>500 cycles), high-rate capability (>1 C), and a stable charge/discharge capacity with minimal overpotential. The ionic conductivity of the GPE was approximately 0.6 × 10^−2^ S/cm at room temperature. Figure 3 illustrates the process of fabricating 3D nanofibrous TiO_2−x_@Li anodes and integrated quasi-solid-state Li-batteries (QSSLBs). The incorporation of the in situ polymerization technique into commercial lamination manufacturing enables the production of 63-Ah pouch cells using LiFePO_4_-graphite. These pouch cells exhibit a high initial Coulombic efficiency, a notable energy density at 0.3 C, an excellent rate capability ranging from 0.25 to 2 C, and long-term cycling performance at 0.3 and 1 C. These results indicate promising prospects for commercial applications and address interfacial issues in lithium-ion batteries, thereby presenting a novel approach for the development of commercially viable Li-metal anodes. [56].

A study by Zhu et al. introduced an innovative approach by incorporating helical mesoporous silica nanofibers (HMSFs) into a PVDF-HFP matrix, resulting in the development of an inorganic polymer gel electrolyte (GPE). This GPE exhibited remarkable characteristics, including a high ionic conductivity of 1.2 × 10^−3^ S/cm, a Li transference number of 0.43, excellent thermal stability up to 372 °C, and a wide electrochemical window of 5.3 V. Furthermore, the researchers also prepared anodes using HMSFs/Fe-N-doped carbon composite nanofibers. The lithium-ion battery (LIB) assembled using the HMSF-based GPE and anode demonstrated a high specific capacity of 1290 mA h/g at 0.3 C, and it exhibited excellent stability over 500 cycles. The presence of HMSFs in both the electrolyte and electrode facilitated effective interphase bridging and the easy transport of Li ions, leading to the enhanced electrochemical performance of the assembled LIB [57]. 

The development of gel polymer electrolytes (GPEs) has shown significant potential for advancing the performance and safety of lithium-ion batteries (LiBs). Various strategies have been explored to overcome the limitations of traditional solid electrolytes, such as their hygroscopic nature, limited chemical stability, and interface resistances. Researchers have successfully utilized alternative precursors, such as LiSnOS, to improve the hygroscopic properties of Li superionic conductor (LISICON)-type solid electrolytes. Additionally, 3D printing techniques have enabled the fabrication of GPEs with precise structures, offering opportunities for the direct printing of electrolytes onto various substrates and the simplification of LiB manufacturing. Furthermore, garnet Li_7_La_3_Zr_2_O_12_ (LLZO) materials with tantalum (Ta) doping have demonstrated promise in reducing interface barriers and improving compatibility in solid-state LiBs. Solid-state electrolytes based on polymeric composites have also emerged as a viable option, offering a high degree of safety and excellent electrochemical performance. Finally, the integration of nanofibrous anodes and highly conductive GPEs has shown potential in enhancing the stability of Li-metal anodes and improving LiB performance. These advancements in GPE technology hold great promise for enabling the development of high-performance and safe LiBs for future applications. 

## 4. Gel Polymer Electrolytes for Lithium Metal Batteries

Lithium metal batteries (LMBs) are highly regarded as potential energy storage devices because of their high theoretical capacity (3860 mA h/g) and low electrochemical potential (−3.04 V). These batteries, with lithium metal as the anode, have found widespread use in various applications such as implantable medical devices, digital watches, and calculators. However, the practical application of LMBs has been hindered by the instability of the lithium metal–liquid electrolyte interface, which is primarily caused by the uncontrolled growth of lithium dendrites. This issue raises serious safety concerns and limits the practical viability of LMBs [58]. Fortunately, solid-state gel polymer electrolytes (GPE) have shown exceptional effectiveness in suppressing the growth of lithium dendrites in LMBs, thereby addressing this critical challenge and paving the way for their practical application. In Table 2, a comprehensive analysis is provided, outlining the comparative aspects of various GPE compositions for Li-metal batteries, including the method of preparation, specific capacity, cyclic stability, and ionic conductivity.

The stability of the lithium (Li) anode and gel electrolyte interface plays a crucial role in improving the coulombic efficiency and cycling performance of lithium-ion batteries (LIBs). In a study by Zuo et al., a gel electrolyte based on a double-polymer network (DPN-GE) was prepared using a single-step photopolymerization process. The researchers employed a solid electrolyte interphase (SEI) strategy to stabilize the interface between the Li anode and the electrolyte. The DPN-GE was formed by creating an interpenetrating network of poly(ether-acrylate) containing silica microspheres as a scaffold and introducing lithium nitrate for the generation of an SEI. The gel electrolyte was composed of poly(ethylene oxide) (PEO), pol(acrylate) (PA), tetraethylene glycol dimethyl ether (TEGDME), and LiTFSI. The DPN-GE exhibited excellent thermal and mechanical stabilities, high room-temperature ionic conductivity, and a stable Li-electrolyte interface. When integrated into quasi-solid-state LIBs, the stable interface provided by the DPN-GE resulted in a dendrite-free morphology and impressive stripping and plating efficiencies. The ionic conductivity of the DPN-GE was measured to be 6.4 × 10^−4^ S/cm at 25 °C, confirming its suitability for ambient temperature battery operation. The LIB utilizing the DPN-GE as an electrolyte showed an initial discharge capacity of 159 mA h/g at 0.1 C rate. At a higher rate of 0.5 C, the capacity remained at approximately 140 mA h/g, and after 100 cycles, 79% of the initial capacity was retained. In contrast, the LIB employing the liquid electrolyte LiTFSI exhibited a rapid capacity decay after only 50 cycles [59].

Organic electrolytes with good wetting characteristics have been preferred for use in lithium-ion batteries (LiBs) due to their ability to form a favorable interface and improve the overall performance. The interfacial chemistry is particularly sensitive to the wetting properties of liquid electrolytes. Ionic liquid-based gel polymer electrolytes (ILGPEs) have shown promise in maintaining suitable viscosity and interface properties compared to common organic liquid electrolytes. In a study by Pan et al., a composite gel polymer electrolyte (ILGPE) based on poly(vinylidene fluoride-co-hexafluoropropylene) (PVDF-HFP) and an ionic liquid was prepared and utilized in LiBs. The ionic liquid used was N-Methyl-N-propylpiperidinium bis(trifluoromethanesulfonyl) imide (PP13TFSI), which was mixed with LiTFSI and dissolved in N-methyl-2-pyrrolidone along with PVDF-HFP. The resulting solution was cast into a film to form the gel polymer electrolyte. When the ILGPE was used, the bulk resistance and ionic conductivity remained unaffected even after 50 cycles of testing. However, when an organic liquid electrolyte was used, significant increases of 50- and 825-fold was observed in the transfer and interfacial resistances, respectively. Wetting the electrode with liquid electrolytes resulted in a maximum discharge capacity of 151.1 mA h/g, while the cells wetted with the ILGPE exhibited a slightly lower discharge capacity of 128.7 mA h/g. However, during cyclic testing, the discharge capacity of the liquid electrolyte-wetted gel polymer electrolyte exhibited a sharp decay, and it eventually short-circuited after 400 cycles. In contrast, the discharge capacities of the ILGPE-wetted cells remained stable even after 400 cycles. The superior performance of the ILGPE can be attributed to the compatibility and stability of the electrode-electrolyte interphase. Furthermore, the ILGPE demonstrated better stability and compatibility with LiFePO_4_ cathodes compared to organic liquid electrolytes [60].

**Figure 4:**
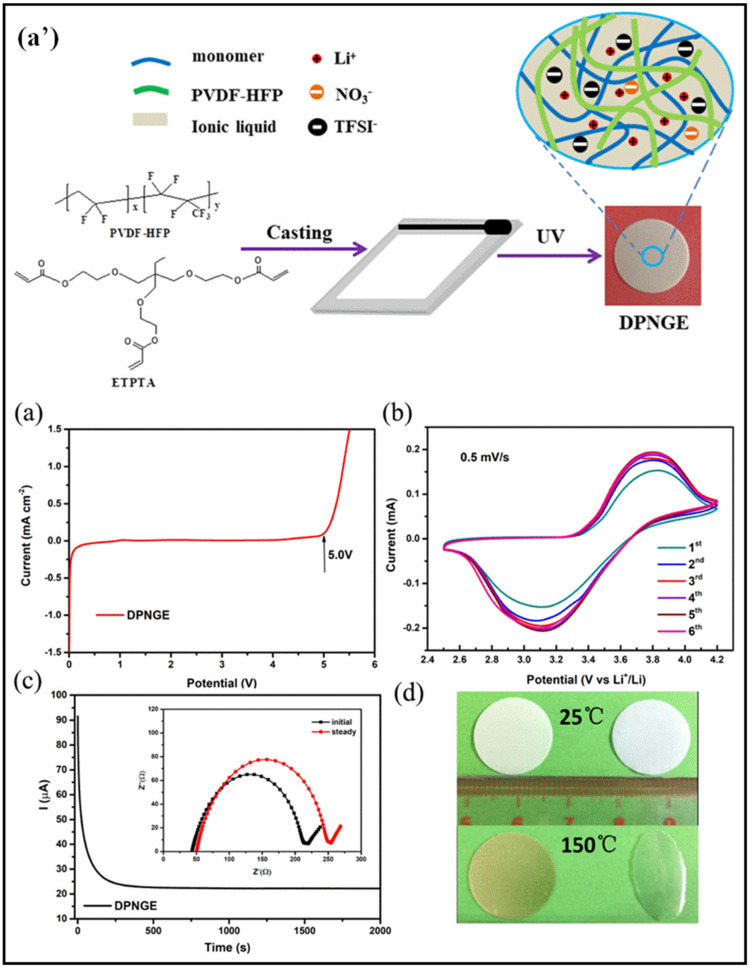
(**a’**) Depicts a diagram that shows the process of creating a gel electrolyte composed of a double-polymer network (DPNGE). The linear sweep voltammetry curve of the DPNGE is illustrated in (**a**), while (**b**) displays the CV curves of the gel electrolyte. (**c**) CA polarization curve and the EIS plot before and after polarization. Finally, (**d**) includes a photograph of the thermal contraction of the DPNGE and the Celgard separator. Reproduced with permission from [61].

The capability of ILs to control the growth of Li dendrites and address interfacial issues is useful for preparing GPEs. ILs offer advantages such as high levels of thermal stability, low viscosities, high levels of room-temperature ionic conductivity, large electrochemical windows, and improved anode stability. Thus, the use of ILs enhances the performance of GPEs and ensures the safety of LIBs. To harness the benefits of ILs, Wu et al. formulated a double-polymer network gel electrolyte (DPNGE) using PVDF-HFP and branched acrylate with the IL Py_13_TFSI. PVDF-HFP was incorporated into the matrix to provide flexibility, while poly(ether-acrylate) (PEA) was selected to form a rigid network through photopolymerization. LiNO_3_ was utilized to create a stable solid electrolyte interphase. The DPGPE facilitated the uniform deposition of Li ions at the interface, enabling a highly reversible plating/stripping process. The process of creating a double-polymer network gel electrolyte (DPNGE) is illustrated schematically in Figure 4a’. The DPNGE exhibits a high ionic conductivity, as evidenced by the LSV curve, and shows good stability under cyclic voltammetry (CV), as demonstrated by the CV curves (Figure 4a,b). The DPNGE is also shown to have good electrochemical stability, as seen in the CA polarization curve and the EIS plot before and after polarization (Figure 4c). A photo is shown of the heat shrink of the DPNGE and the Celgard separator used in the experiment (Figure 4d). The resulting DPGPE exhibited an ionic conductivity of approximately 9.5 × 10^−4^ S cm^−1^ at room temperature, along with an electrochemical window of 5.0 V. When assembled into an LIB, the DPGPE-based battery demonstrated a discharge capacity of 153.7 mA h/g at a 0.5 C rate, with about 92.7% of the initial capacity retained after 500 cycles [61]. 

Solid-state batteries are subject to several disadvantages, including: (i) the breakage of the Li and electrolyte interface due to volume changes during Li anode stripping/plating, (ii) the creation of gaps at the electrolyte–electrode interface, leading to increased electrochemical impedance, and (iii) limited ionic conductivity, resulting in a lower current density than required. To address these issues, Zhu et al. proposed a simple solution using a coiling GPE membrane. They prepared a composite GPE by combining PVDF-HFP and LiTFSI. The composite anode was formed by coiling and cutting stacked GPE films along with Li foil. This vertical alternating array design offers a large electrolyte-Li interface area and enables the continuous transmission of Li ions across the composite anode. The structure also reduces the local current density and resistance at the interface, resulting in more uniform Li stripping/plating. The initial discharge capacity of the cell was 87.5 mA h/g, and after 830 cycles, it decreased to 81.6 mA h/g. The stability of the battery was measured at 93.26%. The GPE demonstrated an ionic conductivity of 3.11 × 10^−5^ S/cm at room temperature [62].

**Figure 5:**
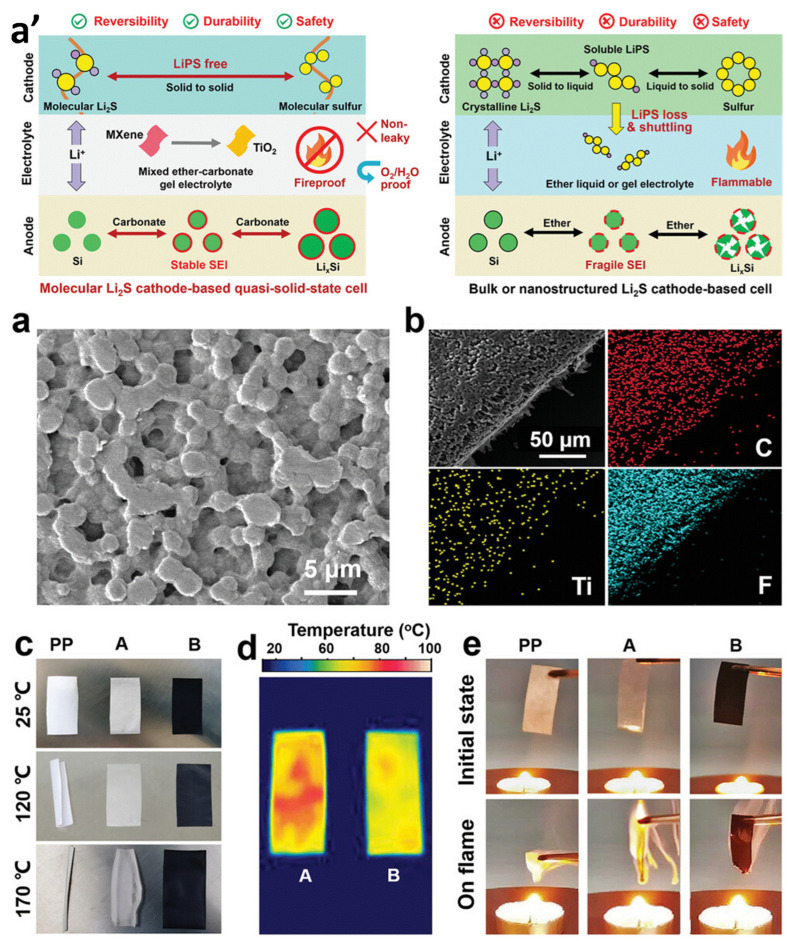
(**a’**) The configuration of a quasi-solid-state lithium battery and highlights its advantages. (**a**) The SEM image of the Mxene-incorporated (M)-PVDF-HFP, while (**b**) shows the elemental mapping of the same film. The thermal resistances of polypropylene (PP), PVDF-HFP (A), and M-PVDF-HFP (B) films during heating are illustrated in (**c**). (**d**) The infrared thermography results that visualize the thermal profiles on the surfaces of PVDF-HFP (A) and M-PVDF-HFP (B) films. Finally, (**e**) the fire-retardancy of the gel electrolyte based on M-PVDF-HFP (B). Reproduced with permission from [63].

High-energy, safe, and cost-effective batteries have garnered significant attention in recent years. One approach to achieving high-energy lithium batteries involves utilizing a Li_2_S molecular cathode and a fireproof gel electrolyte based on solid-state redox chemistry. In a study by Xiangyu Meng et al., a cathode composed of MXene with polyacrylonitrile (PAN)-modified Li_2_S was employed, while silicon (Si) was used as the anode material. The advantages of a quasi-solid-state lithium battery based on the LiPS-free solid-state redox chemistry of a molecular-Li_2_S cathode in a fireproof, mixed ether–carbonate gel electrolyte with MXene as a fire-retardant are illustrated in Figure 5a’. The Mxene-modified (M)-PVDF-HFP film is shown in an SEM image (Figure 5a), and elemental mapping was used to confirm its composition (Figure 5b). The thermal stabilities of polypropylene (PP), PVDF-HFP, and M-PVDF-HFP films were compared upon heating, and infrared thermography was used to visualize the thermal distribution on PVDF-HFP and M-PVDF-HFP films. The M-PVDF-HFP-based gel electrolyte was demonstrated to have superior fire retardancy compared to the PP and PVDF-HFP-based electrolytes, which immediately combusted into flame (Figure 5e). To maximize cell performance, a fireproof gel electrolyte was prepared using Poly (vinylidene fluoride-co-hexafluoropropylene) (PVDF-HFP) and ether-carbonate electrolytes. This gel electrolyte exhibited excellent temperature adaptability, negligible self-discharge, and remarkable reliability. The Li_2_S molecular cathode demonstrated outstanding performance, with a remarkable lifetime of 2000 cycles, an excellent capacity of 830 mA h g/L, 100% coulombic efficiency, and an ultralow capacity loss of 0.005–0.01% per cycle. The battery also exhibited high-rate capability, with achievable rates of up to 10 C. To further enhance the lifetime, cell energy, and compatibility, high-capacity, carbonate-friendly anodes were employed. Looking ahead, the utilization of fire-retardant gel electrolytes has promise for mitigating the risks of short circuits and overheating, and ensuring high levels of safety [63].

To develop high-energy-density lithium metal batteries (LMBs), it is crucial to utilize low-cost and environmentally friendly gel polymer electrolytes (GPEs) that possess wide electrochemical windows, optimal compatibilities, and structural stability. The electrospinning method was employed by Simin Chai et al. to fabricate a three-dimensional biodegradable composite (PAL) composed of Poly-L-lactic acid (PLLA) and poly(acrylonitrile) (PAN) nanofibers. 

The PLLA/PAN nanofiber matrix was then immersed in a mixture of 1,3-dioxolane (DOL) and lithium bis(trifluoromethanesulfonyl)imide (LiTFSI) and subjected to in situ polymerization, resulting in the formation of a gel polymer electrolyte (PDOL-LiTFSI). The formation and strengthening of hydrogen bonds within the PAL composite membrane plays a role in facilitating the migration of Li^+^ ions along the polymer chain. The incorporation of the biodegradable nanofiber membrane led to improvements in the corrosion resistance, ionic conductivity, electrochemical window, and dielectric properties of the gel polymer electrolyte. The cathode material consisted of a LiFePO_4_/PAL composite, while Li metal was used as the anode. The optimized PAN-based gel polymer electrolytes exhibited a high ionic conductivity of 5.17, along with excellent cycling stability of 1000 h at a current density of 0.15 mA/cm^2^ or a charge density of 0.15 mA h/cm^2^. After 140 cycles at a 1 C rate, the LiFePO_4_-based whole cell demonstrated a capacity retention of 97.63%. The utilization of this nanofiber membrane holds significant implications for the design and fabrication of gel polymer electrolytes for lithium metal batteries. Moreover, it contributes to the pursuit of sustainable and environmentally friendly development in energy storage systems [64].

**Figure 6:**
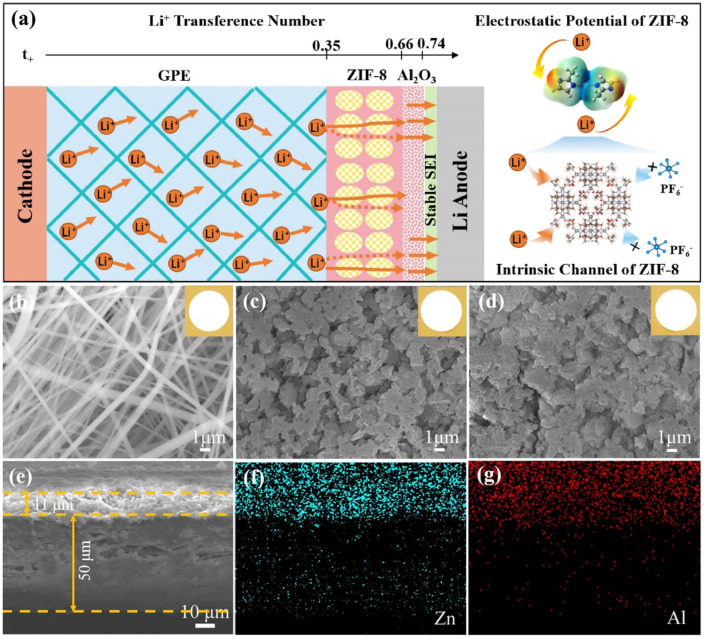
(**a**) An illustrative schematic depicts the heterostructured GPE. (**b**–**d**) SEM images and optical images of the prepared films. (**b**) GPE film, (**c**) GPE-ZIF8 film, and (**d**) GPE-ZIF8-Al_2_O_3_ film. (**e**) SEM image showcases a cross-section of the GPE-ZIF8-Al_2_O_3_ film. (**f**,**g**) The EDS elemental distribution maps are depicted. Reproduced with permission from [66].

Gel polymer electrolytes (GPEs) are highly promising and practical options for portable lithium metal batteries (LMBs). The unique composite design of GPEs allows for the regulation of uncontrolled lithium dendrite growth in liquid electrolytes, as well as the improvement of interface interaction in solid-state electrolytes. A hierarchal layered GPE with a nanofibrous polymer matrix–metal organic framework (MOF)-Al_2_O_3_ composite was utilized by Shaolun Cui et al. To fabricate the nanofibrous matrix, a matrix solution was prepared by dissolving PVDF-HFP, cellulose acetate (CA), and succinonitrile (SN) in a mixture of acetone and *N,N*′-dimethylacetamide. The solution was then electrospun to form the nanofibrous matrix. Subsequently, a dispersion of Z1F8 (zeolite imidazolate framework) nanoparticles and poly(ethylene oxide) (PEO) in anhydrous acetonitrile was solution-casted onto the nanofibrous matrix. Finally, an ultrathin layer of Al_2_O_3_ was deposited using the atomic layer deposition method. Metal organic frameworks (MOFs) were chosen due to their stable skeletons, high specific surface areas, well-defined pore structures, and abundant Lewis acid sites. These properties make MOFs ideal for hosting lithium ions and serving as electrolyte fillers, thereby enhancing ion transport and charge transfer processes while inhibiting the growth of lithium dendrites. As for the anode material, a cathode composed of Mn-based layered oxide with a Li-rich and lithium metal configuration was employed. To ensure uniform lithium-ion interaction and stabilize the interfacial lithium anode, an ultrathin layer of Al_2_O_3_ and the MOF was coated on the same side of the polymer matrix. The incorporation of this composite structure resulted in significant improvements. The transference number of Li^+^ ions increased to 0.74, leading to enhanced cycling stability of over 1000 h. Moreover, the battery retained 84.6% of its initial discharge capacity (257.5 mA h/g at 0.2 C) after 500 cycles. The mechanical strength and interfacial stability were further enhanced by introducing heterostructured ZIF-8 with an ultrathin layer of Al_2_O_3_. Figure 6 illustrates a schematic diagram describing a heterostructured GPE and its varying structure, resulting in an enhanced lithium-ion transference number. It also presents the electrostatic potential distribution and intrinsic channel of ZIF-8. Additionally, the figure displays SEM and optical images of the as-prepared films, in addition to cross-sectional SEM images of the GPE-ZIF8-Al_2_O_3_ films and the EDS mapping results [66].

**Figure 7:**
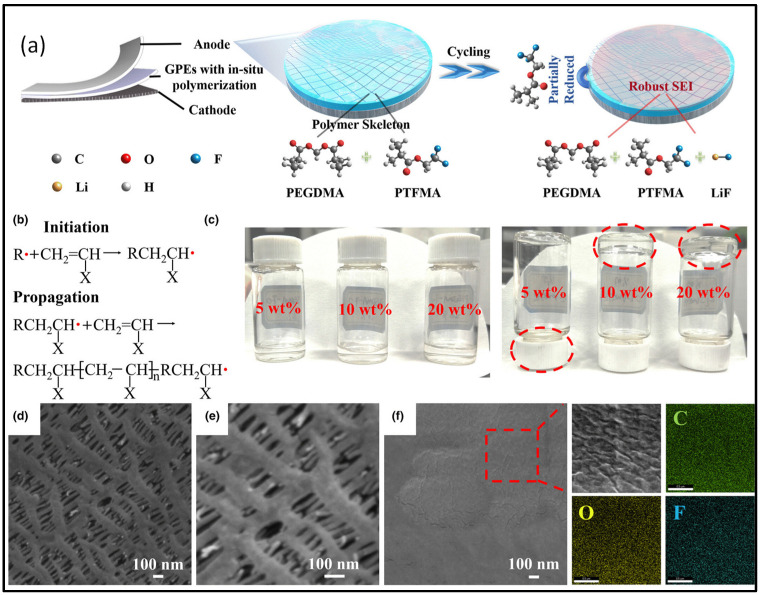
(**a**) A visual representation showcasing the development of lithium anodes within cells featuring in situ GPEs. (**b**) Polymerization mechanism. (**c**) An optical picture exhibits a GPE TFMA featuring different levels of polymer composition. (**d**,**e**) SEM images of the original separator. (**f**) SEM image of the polymer membrane and elemental distribution of carbon (C), oxygen (O) and fluorine (F). Reproduced with permission from [67].

For the development of next-generation power supplies, there is a high demand for quasi-solid-state lithium-metal batteries (LMBs) due to their superior safety and high energy density. To establish these quasi-solid-state batteries, it is essential to introduce fluorine-containing polymer skeletons through in situ gelation. A hybrid framework consisting of poly(ethylene glycol) dimethacrylate (PEGDMA), trifluoroethyl methacrylate (TFMA), and lithium fluoride (LiF) was utilized by Qiyu Wang et al. to prepare a gel polymer electrolyte (GPE) via thermally induced in situ polymerization at 60 °C. The addition of a LiF-enriched solid–electrolyte interphase (SEI) facilitated the growth of dendrite-free Li crystalline grains. The hybrid framework formation resulted in the creation of functional groups, namely -C=C- and -CF_3_. The in situ polymerization reaction led to the formation of the GPE, which exhibited both autogenous stability and facial stability. The anode material employed in this study was Li metal, while LiFePO_4_ was used as the cathode material. By employing this method and utilizing LiFePO_4_ cathodes, a strategy involving the regulation of the -CF_3_ functional group was implemented to achieve a high capacity after 1000 cycles. This strategy resulted in a more stable cycling process, with 90% retention of the initial capacity (170 mA h/g at 0.5 C) after 1000 cycles. Among the trifluoroethyl methacrylate molecules, the reactivity of the -CF functional group (in the order of -CF > -CF2 > -CF3) provided high stability to the anode–electrolyte interface. This contributed to the improved performance and longevity of the battery. Figure 7 shows a schematic representation of the development of lithium anodes in batteries with in situ GPEs. This method of polymerization involves an addition reaction between the main radical R and monomers of PEGDMA and TFMA to create a monomer radical. The liquid electrolyte molecule and these monomer radicals then undergo chain expansion and swelling to form a gel electrolyte. In other words, a sequence of chemical processes that begin with the main radical and monomers and ultimately lead to the development of a gel electrolyte build the polymer skeleton. The picture also shows SEM photographs of the initial separator and polymer membranes as well as an optical shot of a GPE TFMA with a variable polymer composition [67].

Ionic liquids are considered promising due to their advantageous properties such as negligible evaporation, high thermal stability, large potential window, high ionic conductivity, and safety. Zhennan Wang et al. studied the use of LiFePO_4_ as a cathode material, and a gel polymer electrolyte (GPE) was prepared via thermally induced polymerization. The GPE was prepared using the following procedure: a solution of P(VDF-HPF), EMITFSI, PEGDGE, and D-400 (a polyetheramine curing agent) in dimethylformamide (DMF) was mixed. The resulting mixture was solution-casted and subjected to a two-step heating process, with temperatures of 80 °C for 12 h and 120 °C for 24 h. Subsequently, the GPE was dried at 100 °C for 24 h. This GPE exhibited high ionic stability, compatibility, and flexibility. The ionic conductivity of the GPE ranged from 0.05 × 10^−4^ to 1.69 × 10^−3^ S/cm. After 50 cycles at 60 °C, the GPE-based cells showed sustained discharge capabilities of 157 mA h/g. At 0.1 °C and 60 °C, the initial discharge capacity was 162 mA h/g [68].

**Figure 8:**
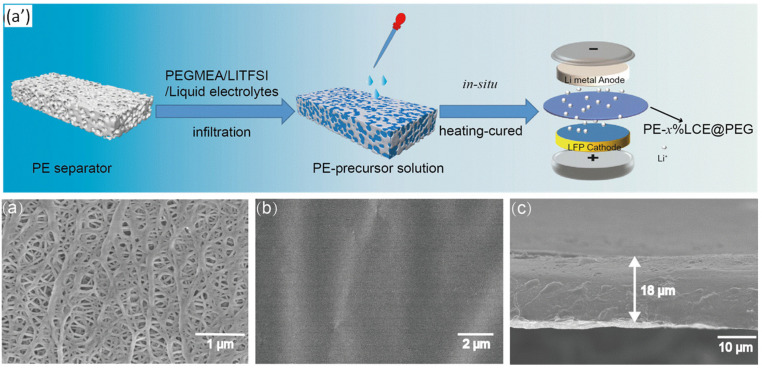
(**a’**) The diagram demonstrates the step-by-step procedure for synthesizing PE-LCE@PEG via in situ polymerization. (**b**) SEM images display the microstructures of (**a**) PE and (**b**) PE-50%LCE@PEG. (**c**) A cross-sectional SEM image provides a detailed view of the PE-50%LCE@PEG. Reproduced with permission from [69].

During the process of in situ polymerization, monomers are mixed with the liquid electrolyte, and gelation occurs as polymerization progresses. The low viscosity of the monomers allows for the good wettability of the GPE by the liquid electrolyte, facilitating well-connected charge transport pathways. In a study by Ma et al., a polyethylene (PE)-based GPE was prepared using in situ polymerization. Figure 8a’ depicts the schematic illustration of the preparation process. A polyethylene glycol methyl ether acrylate (PEGMEA) monomer was used to form the polymer matrix of the GPE. LiTFSI and liquid carbonates were employed as the liquid electrolytes. This mixture was incorporated into a PE separator and polymerized by heating at 60 °C for 12 h. Figure 8a–c displays SEM pictures of the electrolytes generated in polymer gels. When the GPE containing 50% liquid carbonate electrolyte was tested in a lithium-ion battery (LIB), it exhibited a specific capacity of 158.5 mA h/g at a rate of 0.5 C. After undergoing 100 cycles, 98.87% of the initial capacity was retained, indicating good cycle stability. The PEG component showed excellent compatibility within the system. Furthermore, this GPE demonstrated good chemical stability and high ionic conductivity, especially at elevated temperatures. The GPE containing 50% liquid carbonate electrolyte enabled the continuous illumination of a yellow LED in various states, including the flat state, folded state, several folds, and even after being cut. The maximum ionic conductivity exhibited by the PE-GMEA-GPE was 1.73 × 10^−4^ S/cm at 60 °C. These findings highlight the reliable performance and safety of using this GPE in LIBs [69].

Solid-state Li-Te batteries have drawn significant interest because of their excellent performance and security. In order to develop quasi-solid-state Li-Te batteries, Yue Zhang and colleagues used a gel polymer electrolyte (GPE), Li metal as the anode, and Te/C as the cathode. A comparison was also made with S/C and Se/C cathodes in which the Te/C exhibited superior electrochemical performance. The gel polymer electrolyte (GPE) was fabricated via a solution-casting technique by blending poly(vinylidene fluoride-co-hexafluoropropylene) (PVDF-HFP) and N-methyl-2-pyrrolidone (NMP) in a 1:4 ratio. The cathode material was prepared using a mixture of Te/C composite in a 2:1 ratio. S/C and Se/C cathodes were prepared using a mixture in a 1:1 ratio. The PVDF-HFP polymer in the GPE provided thermal and electrochemical stability, a suitable dielectric constant, and mechanical strength. The GPE exhibited a high ionic conductivity of 8.0 × 10^−4^ S cm^−1^ at 25 °C and a wide electrochemical window of up to 4.82 V. The outstanding interfacial stability of Te in Li-Te batteries contributed to good rate capability and high electrical conductivity. This resulted in superior electrochemical performance compared to S/C and Se/C cathodes [70].

Deep eutectic solvents (DES) are being developed as potential electrolytes for lithium-ion batteries (LiBs) due to their low cost, low vapor pressure, high electrochemical stability, superior flame retardance, and properties similar to ionic liquids (IL). However, modifications of DES-based gel polymer electrolytes (GPE) are necessary to attain high a specific capacity and stability. A DES-based GPE with a rapid self-healing capability was prepared by Qiqi Li et al. via the in situ copolymerization of pentaerythritol tetraacrylate (PETEA), cystine, and 2-isocyanoethyl methacrylate (ICEM). This GPE demonstrated the ability to self-smooth cracks within 30 min. The resulting GPE exhibited a high ionic conductivity of 1.1 × 10^−3^ S/cm at 25 °C. Moreover, it displayed a large potential window of 4.5 V, stability for over 1200 h, and a high specific capacity of 135.4 mA h/g. By combining a lithium metal anode with a LiFePO_4_ cathode and the DES-based GPE, high-energy-density and fire-resistant LiBs were achieved. These findings highlight the use of DES-based electrolytes in the design of advanced lithium-ion batteries [71].

GPEs have the potential to significantly enhance the performance, stability, and safety of LMBs. The utilization of double-polymer network (DPN-GE) gel electrolytes, interpenetrating networks, and SEI strategies has resulted in stable Li-electrolyte interfaces, dendrite-free morphologies, and enhanced stripping/plating efficiencies. Ionic liquid-based gel polymer electrolytes (ILGPEs) have demonstrated superior wetting properties, compatibility, and stability, leading to improvements in the interfacial chemistry and cycling performance. Incorporating organic electrolytes with high thermal stabilities and low viscosities, as well as employing coiling GPE membranes and fireproof gel electrolytes, has addressed interfacial issues, enhanced anode stability, and facilitated continuous Li ion transmission. Additionally, the utilization of biodegradable nanofiber membranes has improved corrosion resistance, ionic conductivity, and cycling stability in GPEs. 

The utilization of composite designs in GPEs, such as the nanofibrous polymer matrix-metal organic framework (MOF)-Al_2_O_3_ composite, has allowed for effective regulation of lithium dendrite growth and enhancement of interface interaction. By incorporating stable MOFs with high surface areas and Lewis acid sites, ion transport and charge transfer processes are improved while inhibiting dendrite formation. Additionally, the introduction of fluorine-containing polymer skeletons through in situ gelation has enabled the development of quasi-solid-state LMBs with enhanced safety and capacity retention. The utilization of ionic liquids as next-generation electrolytes has shown advantageous characteristics, including wide electrochemical windows, high ionic conductivity, and high thermal stability. In situ polymerization techniques have been employed to create gel polymer electrolytes (GPEs) with excellent ionic stability, compatibility, and flexibility. Moreover, the combination of gel polymer electrolytes with specific anode and cathode materials, such as Li metal and LiFePO_4_, has resulted in significant improvements in cycling stability and capacity retention. These advancements in GPE technology contribute to the development of high-energy-density, safe, and environmentally friendly lithium batteries and hold promise for future applications in the field of energy storage.

## 5. Lithium–Oxygen Batteries

Similar to lithium-ion batteries (LIBs), lithium metal is used as an anode in lithium–oxygen batteries (LOBs). In LOBs, a conducting porous matrix is employed as the cathode. The anode undergoes Li metal dissolution, while oxygen reduction/oxygen evolution takes place at the cathode. LOBs offer high operational voltages (∼2.96 V) and formal specific energies (∼3500 Wh/kg). To address the performance and safety concerns common to LIBs, gel polymer electrolytes (GPEs) with high levels of ionic conductivity and interconnected polymer networks have been utilized. GPEs play a vital role in enhancing the performance of LOBs by preventing Li metal corrosion and electrolyte evaporation. Recently, GPEs incorporating inorganic fillers and polymer matrixes with high thermal and mechanical stabilities, as well as biodegradable polymers, have been developed for LOBs. These advancements aim to improve the overall efficiency, safety, and lifespan of LOBs [72,73]. Table 3 presents a comprehensive overview of different GPE compositions employed in Li-oxygen batteries, examining key factors such as the preparation method, specific capacity, cyclic stability, and ionic conductivity.

To safeguard the lithium anode from electrolyte evaporation, a hybrid solid–gel electrolyte was investigated by Wen-Bin Luo et al. for use in lithium batteries (LiBs). The GPE was prepared by coating RuO_2_-graphene oxide composites onto nickel foam as the cathode, and a monomer mixture capable of forming a gel via in situ polymerization was applied by brushing. The monomer mixture consisted of tetraethylene glycol dimethyl ether, ethoxylated trimethylolpropane triacrylate (ETPTA), and 2-hydroxy-2-methyl-1-phenyl-1-propanone. This hybrid solid–gel electrolyte served as both the separator and the electrolyte in the LiB. After 140 cycles at a current density of 0.4 mA/cm^2^, the flexible LiB demonstrated a terminal voltage of 2.2 V and a capacity of 1000 mA h/g. The solid–gel electrolyte exhibited a lower activation energy and higher ionic conductivity compared to typical liquid electrolytes. Furthermore, this solid–gel electrolyte effectively protected the lithium metal anode during long-term cycling processes [76].

GPEs have found applications in lithium–oxygen batteries, which are well-known for their high energy densities. Among the various options, PVDF-HFP-based GPEs filled with different lithium salts have been preferred over liquid electrolytes. In a study conducted by Celik et al., the effects of different lithium salts on GPE performance, including ionic conductivity, cyclic stability, electrochemical stability, and interfacial stability, were investigated. Specifically, lithium perchlorate (LiClO_4_), lithium bis(trifluoromethanesulfonyl)imide (LiTFSI), and lithium hexafluorophosphate (LiPF_6_) were examined. The results showed that LiPF_6_ exhibited the highest ionic conductivity (8 × 10^−5^ S/cm) compared to LiClO_4_ (3.7 × 10^−5^ S/cm) and LiTFSI (3 × 10^−5^ S/cm). However, LiTFSI demonstrated the highest Li ion transference number (0.77) and the widest electrochemical window (4.9 V). Additionally, the cyclic stability of LiTFSI was four times higher than that of the other lithium salts studied. The highest overall ionic conductivity was observed in the PVDF-HFP/LiPF_6_ system, reaching 9.44 × 10^−5^ S/cm. This enhanced conductivity can be attributed to the smaller size of the LiPF_6_ anions [35].

**Figure 9:**
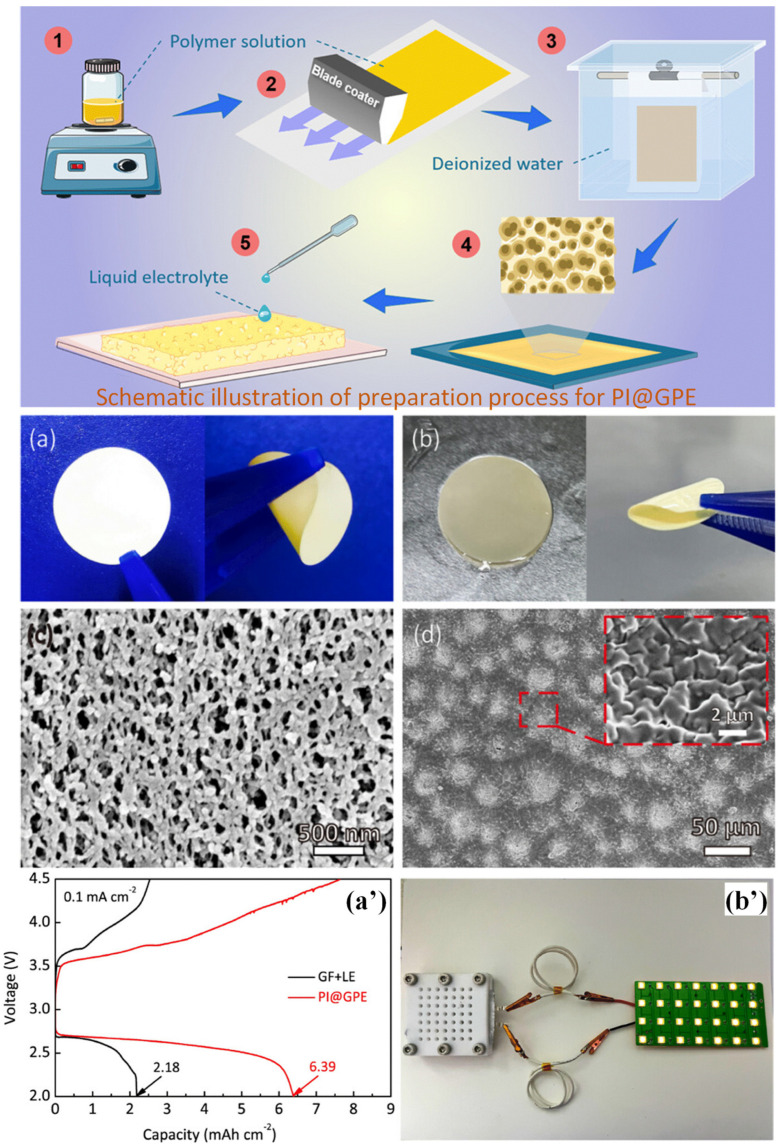
Preparation procedure of porous polyimide membrane and subsequent PI@GPE (1) dissolving polyimide powder in DMF. (2) Pouring the obtained polyimide solution on a glass substrate to form a uniform liquid film by the blade. (3) Conducting phase inversion by immersing the glass substrate into deionized water, resulting in the formation of a porous polyimide membrane. (4) Removing residual solvents through a thorough drying procedure to obtain a cross-linked porous polyimide membrane. (5) Swelling and gelling the polyimide membrane in the liquid organic electrolyte to acquire the final PI@GPE. Flat and curved optical images of (**a**) polyimide and (**b**) PI@GPE. SEM of (**c**) polyimide and (**d**) PI@GPE; (**a’**) Lithium–air pouch cell discharge capabilities with a 2 V end-of-discharge voltage default. (**b’**) Optical image of a lithium–air battery powering light-emitting diodes at PI@GPE bases. Reproduced with permission from [74].

Polyimide (PI) is a highly durable polymer known for its exceptional thermal and structural stability. In the context of gel polymer electrolytes (GPEs), PI-based cross-linked formulations have demonstrated impressive thermal stability, withstanding temperatures of up to 334 °C. Moreover, these GPEs exhibit a remarkable organic liquid electrolyte uptake of 178%. Leveraging these properties, Xu et al. developed a PI-based GPE to boost lithium–oxygen battery cycle performance. Figure 9 shows the schematic illustration of the preparation process. The fabrication process involved the preparation of a PI membrane through the use of a solution of PI in *N,N*′-dimethylformamide via a phase inversion method, wherein deionized water served as the non-solvent. Subsequently, the PI membrane was immersed in a mixture of liquid electrolytes, namely LiTFSI, lithium bromide, and tetra(ethylene glycol) dimethyl ether, to obtain the final PI GPE. Figure 9a,b show optical photographs of the polyimide membrane and PI@GPE (polyimide with gel polymer electrolyte), correspondingly, whereas Figure 9c,d show pictures of the membrane and PI@GPE, obtained via SEM. This GPE formulation exhibited notable characteristics, including a high ionic conductivity (4.4 × 10^−4^ S/cm) and a lithium transference number of 0.596. Upon its integration into a lithium–oxygen battery, the PI GPE demonstrated remarkable cyclic stability, enduring up to 366 cycles at a current density of 0.1 mA. The discharge capabilities of a lithium–air pouch cell with a default end-of-discharge voltage of 2 V are shown in Figure 9a’. Figure 9b’ shows an optical image of a lithium–air battery powered by PI@GPE that powers light-emitting LEDs. The interior structure of the PI GPE exhibited a compact and homogeneous nature, enabling a stable flow of lithium ions while effectively suppressing dendrite growth [74]. 

**Figure 10:**
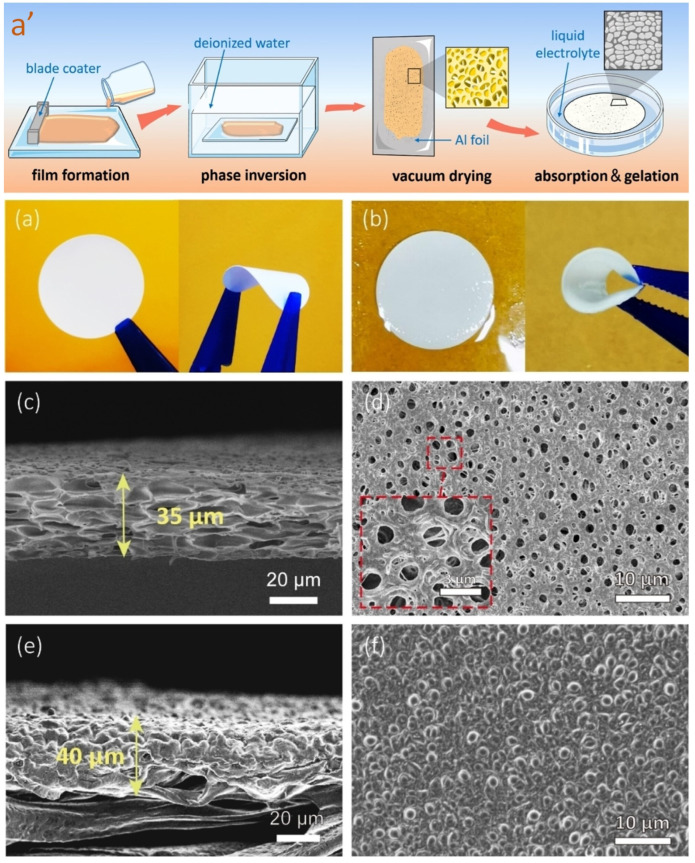
(**a’**) Synthesis procedure for CA@GPE. Optical photos of (**a**) the porous cellulose acetate membrane (PCM) and (**b**) the CA@GPE. SEM images: (**c**) cross-section and (**d**) top of the PCM; (**e**) cross-section and (**f**) top surface of the CA@GPE. Reproduced with permission from [75].

Despite facing challenges such as electrolyte evaporation, lithium–oxygen batteries have garnered significant attention due to their exceptionally high theoretical power density, making them a promising candidate among secondary batteries. The anode material consists of lithium foil, while foamed nickel serves as the cathode current collector. In the pursuit of developing a gel polymer electrolyte (GPE) for these batteries, a combination of cellulose acetate (CA) and poly(vinylpyrrolidone) was dissolved in NMP. The resulting solution was cast onto a glass plate and subjected to phase inversion by immersing it in water. Subsequently, the CA film was immersed in a solution of lithium bis(trifluoromethanesulfonyl)imide (LiTFSI) to obtain the final CA-based GPE. Figure 10a’ provides a visual representation outlining the steps involved in the synthesis process, in addition to optical photos and SEM images of the membranes. The CA-GPE exhibited excellent cyclic stability for an extended period of up to 1200 h. This GPE formulation offered a broader potential of 5.0 V and displayed a high lithium-ion transference number of 0.595. Notably, CA is an inexpensive, biodegradable, and renewable material that possesses the unique property of forming hydrogen bonds with TFSI-, thereby facilitating the dissociation of the lithium salt. Additionally, the C=O groups present in CA play a significant role in removing electrons and promoting the movement of lithium ions by limiting the mobility of TFSI- [75].

The development of hybrid solid-gel electrolytes and gel polymer electrolytes (GPEs) has made significant progress in developing the high performance and stable lithium–oxygen batteries. The hybrid solid–gel electrolyte composed of RuO_2_-graphene oxide composites and a gel-forming monomer mixture serves as both the separator and electrolyte, effectively protecting the lithium metal anode and demonstrating excellent cyclic stability. Similarly, GPEs based on poly(vinylidene fluoride)-based matrixes, super-high ionic conductive gel polymers (SHGPs), and polyimide (PI) have shown significant improvements in ionic conductivity, cyclic stability, and interfacial stability. These GPEs enable enhancements to the performance and longevity of lithium–oxygen batteries, offering higher specific capacities, improving voltage retention, and extending the cycling life. The choice of lithium salts in GPEs also influences their performance, with lithium bis(trifluoromethanesulfonyl)imide (LiTFSI) demonstrating superior characteristics, including a wide electrochemical window and high transference number. 

Moreover, the incorporation of renewable and biodegradable materials, such as cellulose acetate (CA), adds sustainability and cost-effectiveness to the GPE formulations. Collectively, these advancements in GPE technology contribute to the advancement of lithium battery systems and enable the creation of effective and dependable energy storage systems.

## 6. Lithium–Sulfur Batteries

In lithium–sulfur batteries (LIS), the power generation process involves the gradual transition of sulfur through charge and discharge cycles. The use of elemental sulfur, a byproduct of the petrochemical industry, as the cathode material in LIS significantly reduces the cost compared to lithium-ion batteries (LIBs). Theoretically, LIS has a high energy density of 2600 Wh/kg and a specific capacity of 1675 mA h/g. However, there are several challenges associated with LIS that must be addressed to ensure their safe and efficient operation. One of the main issues in LIS is the volume expansion of sulfur during cycling, which can lead to mechanical stress and electrode degradation. Furthermore, the development of lithium dendrites on the anode surface raises safety issues since they may result in short circuits and even thermal runaway. These challenges can be mitigated by incorporating a uniform charge distribution and utilizing the flexibility of gel polymer electrolytes (GPEs). GPEs provide a stable and conductive environment for the movement of lithium ions, reducing the likelihood of dendrite formation and enabling safer battery operation. A further issue that is addressed by the usage of GPEs in LIS is the capacity fading issue brought on by repeated charge and discharge cycles. GPEs can effectively encapsulate the active sulfur material and provide a stable interface between the cathode and electrolyte, preventing the loss of active material and maintaining consistent electrochemical performance over time [77]. A comparison of GPE compositions for Li-sulfur batteries is displayed in Table 4, covering aspects such as the preparation method, specific capacity, cyclic stability, and ionic conductivity.

In order to enhance the interface between lithium (Li) and gel polymer electrolytes (GPEs), lithiophilicity has been introduced to improve the stability of the Li anode during cycling in lithium–sulfur (Li-S) batteries. Han et al. employed poly(dopamine) (PDA) modification on a polyvinylidene fluoride (PVDF)-based GPE to impart lithiophilicity. The lithiophilic nature of PDA, which contains pyrrolic nitrogen, facilitates Lewis acid–base interactions. Solution casting was first used to prepare PVDF film, followed by coating both sides of the PVDF film with dopamine, which underwent oxidative polymerization to form PDA. The resulting PVDF-PDA film was then infiltrated with a mixture of liquid electrolyte containing lithium bis-(trifluoromethane)sulfonimide (LiTFSI), 1,3-dioxolane, and 1,2-dimethoxyethane (DME). The strong confinement of polysulfide species by the polydopamine layer provided improved the interfacial stability of the Li anode and increased the cyclic stability of the Li-S battery. The quasi-solid-state cell with the polydopamine-modified GPE exhibited an extended cycling life. The specific capacity demonstrated by the PVDF-PDA GPE was 1215.4 mA h/g at a current rate of 0.1 C. After 200 cycles, the initial specific capacity decreased to 868.8 mA h/g, corresponding to a decay of approximately 0.14% per cycle. The stability after 200 cycles was 71.48%. In comparison, the specific capacities exhibited by cells with PVDF and Celgard films were 1164.58 mA h/g and 1186.8 mA h/g, respectively. After 200 cycles, the respective initial specific capacities of the PVDF and Celgard films decreased to 733.6 mA h/g and 509.7 mA h/g. The PVDF-PDA GPE demonstrated an ionic conductivity of 5.71 × 10^−4^ S/cm at room temperature, which is higher than those of the GPEs made using Celgard (3.03 × 10^−4^ S/cm) and PVDF (4.51 × 10^−4^ S/cm). Therefore, the PDA modification of PVDF was found to be effective in improving the performance of Li-S batteries, particularly in terms of cyclic stability and ionic conductivity [78].

**Figure 11:**
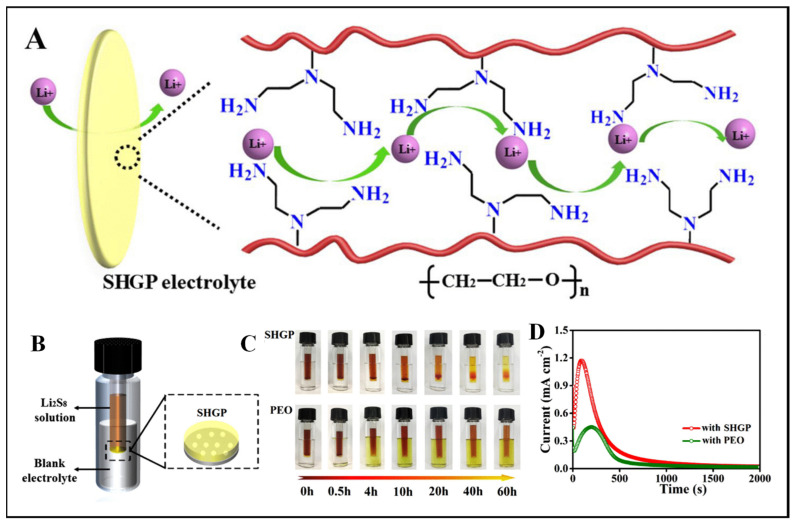
(**A**) The schematic diagram and operational concept of the SHGP gel electrolyte with a high level of ionic conductivity. (**B**) A visual experimental setup is depicted in which the blank electrolyte consists of a 1 M LiTFSI solution in a 1:1 volume ratio of DOL and DME. (**C**) A visual comparison is shown to evaluate the adsorptivity of Li_2_S_8_ to both SHGP and PEO. (**D**) Potentiostatic discharge curves at 2.08 V are plotted to illustrate the Li_2_S deposition process using an SHGP and PEO as electrolytes, respectively. Reproduced with permission from [79].

To address the challenges of Li_2_S deposition and lithium dendrite formation caused by repeated stripping/plating and the shuttle effect resulting from soluble polysulfides in lithium–sulfur (Li-S) batteries, a super-high ionic conductive gel polymer (SHGP) electrolyte was developed by Zhou et al. Figure 11A shows a schematic representation of the SHGP’s operation. The SHGP electrolyte has a molecular structure that includes polar nitrogen heteroatoms and ether chains. These functional groups facilitate the coordination and transport of lithium ions within the electrolyte. The presence of these polar functional groups enables them to interact and coordinate with lithium ions, promoting their mobility. The SHGP electrolyte was prepared by polymerizing a mixture of poly(ethylene glycol)/diglycidyl ether (PEGDE), branched polyethylenimine (PEI), and Li bis(trifluoromethanesulfonyl)imide (LiTFSI). The mixture was then cast into a film. The ether chains in the electrolyte exhibited strong interactions with the Li ions, promoting effective Li transport. The lone pairs on the amine nitrogen atoms crosslinked the polymer via Li-ion coordination. These interactions between the SHGP and the Li ions facilitated a high level of Li ion conductivity. Figure 11D shows the potentiostatic curves; when comparing a cell using the SHGP electrolyte to one using a PEO (polyethylene oxide) electrolyte, the SHGP cell shows a significantly stronger current peak. This suggests that the SHGP electrolyte has a higher affinity for Li_2_S_8_ (lithium octasulfide) and facilitates the efficient deposition of Li_2_S. This enhanced performance is attributed to the presence of abundant nucleation sites within the SHGP electrolyte, which are derived from its enriched polar functional groups. The SHGP electrolyte demonstrated an ionic conductivity of 2.2 × 10^−3^ S cm^−1^ at 60 °C and 0.75 × 10^−3^ S cm^−1^ at 30 °C. The Li-S battery with the SHGP electrolyte had a specific capacity of 950 mA h/g at 0.2 C, and it maintained 98% of its original capacity after 100 cycles. Theoretical simulations verified that the polar functional groups of SHGP interacted with lithium polysulfides, preventing their migration and hindering their detrimental effects. When tested in quasi-solid-state Li-S batteries, the SHGP electrolyte exhibited improved performance, maintaining its stability over 400 cycles [79].

The development of GPEs has proven effective in addressing key challenges in lithium–sulfur (Li-S) batteries. Enhancing the interfaces between lithium (Li) and GPEs has been achieved by introducing lithiophilicity, as demonstrated via the poly(dopamine) (PDA) modification of a polyvinylidene fluoride (PVDF)-based GPE. The lithiophilic nature of PDA enhances the interfacial stability and cyclic stability of Li-S batteries, resulting in an extended cycling life and improved specific capacity. The quasi-solid-state cell with a polydopamine-modified GPE exhibited a high specific capacity and stability after 200 cycles. Furthermore, the development of a super-high ionic conductive gel polymer (SHGP) electrolyte addresses the challenges of Li_2_S deposition, lithium dendrite formation, and the shuttle effect. The SHGP electrolyte demonstrates excellent Li ion conductivity and exhibits a high specific capacity, retaining its stability over a significant number of cycles. These advancements in GPE technology contribute to the overall performance enhancement and viability of Li-S batteries, offering a promising solution for next-generation energy storage systems.

## 7. Zinc Batteries

Zinc-based batteries are emerging as next-generation energy storage technologies with significant potential, finding applications in wireless devices, hearing aids, and signal lamps. Unlike lithium-based batteries, zinc batteries do not require stringent dry room conditions for their operation. Moreover, zinc is abundantly available in the Earth’s crust and can be easily recycled, resulting in lower manufacturing costs. The The required changes have been adopted. of gel polymer electrolytes (GPEs) in zinc batteries has shown great potential, particularly in wearable electronics in which the ability to withstand mechanical deformation is crucial. GPEs play a significant role in influencing the power output and cyclic stability of zinc batteries. Therefore, the rational design of GPEs becomes essential in achieving superior mechanical stability and accelerated ion transport. Recent years have seen significant efforts made in developing GPEs tailored for various types of zinc batteries. The primary objective is to enhance mechanical stability, ionic stability, mechanical stability, and cyclic stability, thereby optimizing the overall performance of zinc batteries [80,81]. Table 5 presents a comprehensive overview of different GPE compositions employed in zinc batteries, examining key factors such as the preparation method, specific capacity, cyclic stability, and ionic conductivity.

Zinc–air batteries (ZABs) have gained significant interest as potential substitutes for LIBs. However, their development has been hindered by challenges related to their low energy conversion efficiency and output power density, especially in flexible ZABs. To address these limitations, a polyacrylamide (PAM)-based alkaline gel electrode (PAM-AGE) was utilized in this study. The PAM-AGE demonstrated a high hydroxide ion conductivity, reaching 0.2156 S cm^−2^, which is advantageous for enhancing battery performance. By incorporating the PAM-AGE, the power density of the ZAB was significantly improved, reaching up to 105.0 mW cm^−2^. This enhancement in power density is a noteworthy achievement in the development of flexible ZABs [82].

The safety and affordability of aqueous zinc-ion batteries (AZIBs) is well known. However, their industrial applications are limited due to the high freezing point of the electrolyte. To address this issue, Zhou et al. developed a polyvinyl alcohol–glycerol (PVA/G) gel polymer electrolyte (GPE) for AZIBs. The PVA/G GPE was prepared by mixing an aqueous solution of polyvinyl alcohol and glycerol, followed by solution casting. The PVA/G GPE was sandwiched over a zinc foil and a titanium foil, with d-MgVO serving as the active material, to create the AZIB. Remarkably, the assembled AZIB exhibited excellent performance over a wide range of temperatures (from −30 to 60 °C). The ionic conductivity of the PVA/G GPE was measured at different temperatures, with values of 22.4, 18.2, 14.3, and 10.7 mS/cm at 60, 25, 0, and −30 °C, respectively. The AZIB also demonstrated a volumetric energy density of 79.35 mW h/cm^−3^. The discharge capacity reached 406.4 mA h/g at a rate of 0.2 C, and the battery showed remarkable stability, with a capacity retention of 96% after 300 cycles. Notably, this battery exhibited heat resistance and excellent mechanical stability. The PVA/G electrolyte demonstrated environmental adaptability and suitability for practical applications [83]. 

**Figure 12:**
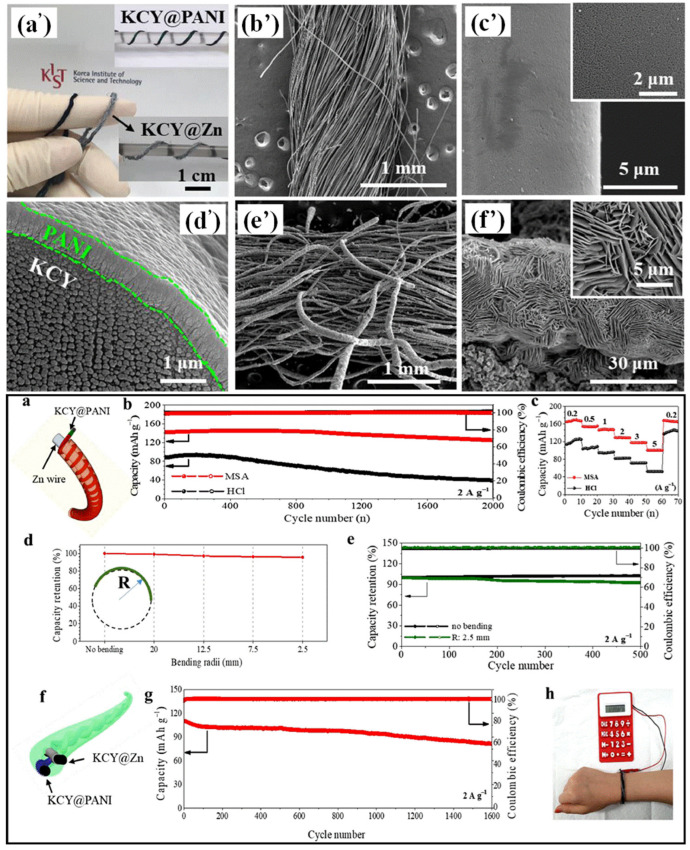
(**a’**) The optical images present the electrodes based on KCY materials, namely KCY@PANI and KCY@Zn. KCY, or Kynol Novoloid carbonized yarn, is a flexible material utilized in conjunction with a gel polymer electrolyte doped with MSA (methanesulfonic acid) to improve the flexibility of aqueous Fs-ZPBs (fiber-shaped zinc-polymer batteries). (**b’**,**c’**) FE-SEM images provide a top-view perspective of KCY@PANI at different scales. (**d’**) Cross-sectional FE-SEM images demonstrate the embedding of KCY@PANI in epoxy resin. (**e’**,**f’**) FE-SEM images depict the top-view of the KCY@Zn at different scales. (**a**) Schematics are employed to illustrate the structure of Fs-ZPB featuring a Zn wire. (**b**) The cycling stability of Fs-ZPBs at a specific current of 2 A g^−1^ and within a potential of 0.7–1.5 V is presented. (**c**) The rate capabilities with Zn wire anodes are evaluated. (**d**) The capacity retentions of bent Fs-ZPBs with various curvature radii are displayed. (**e**) The cycling performance evaluation under a curvature radius of 2.5 mm (**f**) Schematics of fiber-shaped all-carbon ZPBs. (**g**) The cycling performances of all-carbon ZPBs. (**h**) An optical photo showcases an all-carbon Fs-ZPB effectively powering a calculator. Reproduced with permission from [84].

Methanesulfonic acid (MSA) is a commonly used protonating agent for poly(aniline) (PANI) due to its favorable properties, such as its high ionic conductivity, low toxicity, and solubility in water. It plays a crucial role in achieving mechanically and thermally stable PANI with improved conductivity. MSA effectively localizes the charge carriers within the PANI chains, thereby enhancing its overall performance. The interaction between the MSA and the amine/imine groups in protonated PANI forms hydrogen bonds, contributing to enhanced cyclic stability. In the context of fiber-shaped Zn-PANI batteries (Fs-ZPBs), there are certain limitations to be addressed, including low levels of electrolyte conductivity, soluble quinone formation, and capacity fading. To overcome these challenges, Shim et al. utilized MSA to enhance the ionic conductivity of polyvinyl alcohol (PVA)-based gel polymer electrolytes (GPEs). The incorporation of MSA into the PVA matrix resulted in remarkable stability of the GPE, with a capacity retention of 88.1% after 2000 cycles and 92.7% after 500 bending cycles (radius = 2.5 mm). The initial capacity of the battery was measured at 100.3 mA h/g with a 2C rate. The intermolecular hydrogen bonds formed between the PVA and MSA played a crucial role in connecting the PVA molecular chains and PANI chains on the surface of the PVA. These linkages significantly improved the charge transfer properties and ionic conductivity within the battery system. Furthermore, the coordination of large sulfonate ions with PANI effectively prevented the binding of water molecules to the PANI surface, which is a major cause of PANI degradation. This protective effect of MSA on the PANI surface further contributes to the enhanced stability and overall performance of the battery. Figure 12 provides visual illustrations and characterization data supporting these findings. Optical images of KCY-based electrodes, KCY@PANI and KCY@Zn, demonstrate the flexibility achieved through the association of Kynol Novoloid carbonized yarn (KCY) with a gel polymer electrolyte doped with MSA. FE-SEM images provide insights into the morphologies and structural features of the KCY@PANI cathode and KCY@Zn anode. The cycling performances, rate capabilities, capacity retentions under bending, and long-term cycling are evaluated and presented, showcasing the excellent electrochemical performance and flexibility of the fiber-shaped all-carbon ZPBs [84].

**Figure 13:**
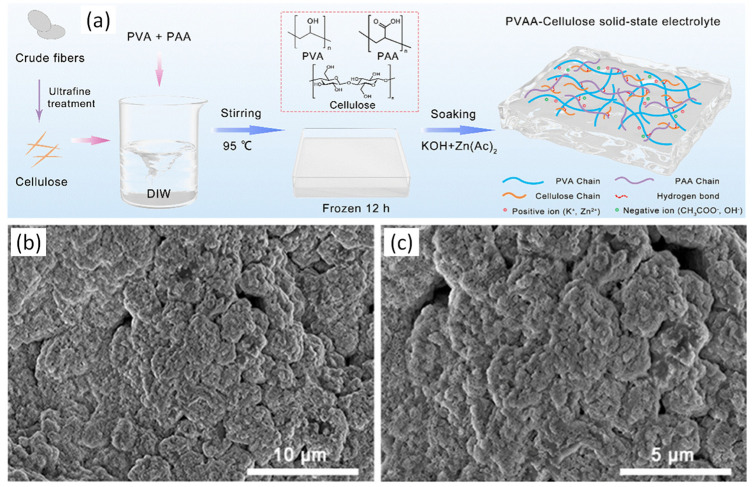
(**a**) The synthesis procedure for the poly(vinyl alcohol) and poly(acrylic acid) (PVAA)-cellulose solid-state electrolytes (SSEs) is presented. (**b**) SEM images showcase the PVAA-cellulose SSE. (**c**) A magnified picture highlights the PVAA-cellulose SSEs. Reproduced with permission from [85].

The advancement of high-performance, safe, and flexible GPEs is crucial for zinc–air batteries (ZABs). However, poly(vinyl alcohol) (PVA)-based GPEs suffer from limitations such as poor mechanical strength, low ionic conductivity, and short cyclic stability. To overcome these challenges, Li et al. devised a novel approach by preparing a multinetwork cross-linked composite GPE. The composite GPE, named PVAA-cellulose, was created by incorporating poly(acrylic acid) (PAA) and ultrafine cellulose into the PVA gel matrix. Figure 13 illustrates the synthetic schematic and SEM images of the prepared solid-state electrolytes. Cellulose, with its abundant hydroxyl groups, was able to improve the mechanical strength and specific surface area of the cross-linked composite GPE. The PVAA-cellulose exhibited optimal water retention, thermal stability, and a high ionic conductivity of 0.123 S/cm. Moreover, it effectively suppressed dendrite growth and the generation of oxidation byproducts on zinc anodes, leading to improved cycling stability for the ZABs. The PVAA-cellulose electrolyte-assembled solid-state ZABs showed outstanding performance characteristics. They exhibited a high power density, reaching 74 mW/cm^2^, and a specific capacity of 724 mA h/g at a current density of 3 mA/cm^2^. Additionally, the cyclic stability of the batteries was maintained for over 56 h. The PVAA-cellulose displayed remarkable mechanical strength, with values of 87 MPa and 818% elongation, which could be due to the formation of an intertwined network structure [85].

In an effort to enhance the efficiency of quasi-solid-state zinc–air batteries (QSZABs), Li et al. explored the use of hollow Sn microspheres modified with 2-hydroxypropyl-β-cyclodextrin (hollow Sn-inner HPβCD) to regulate alkali release within the GPE. The incorporation of hollow Sn-inner HPβCD proved to be highly effective in preventing alkali leakage, facilitating alkali diffusion back to the GPE during the charging process, and reducing the loss of soluble Zn(OH)_4_^2−^. As a consequence, the QSZAB’s performance greatly increased, and a long cyclic lifespan of 127 h was achieved. This research provides valuable insights into enhancing the performance of QSZABs by regulating alkali release at the GPE-anode interface [86].

The development of GPEs has emerged as a hopeful avenue for advancing the performance of zinc-based batteries. These batteries offer several advantages such as lower manufacturing costs, the abundant availability of zinc, and the ability to operate without stringent dry room conditions. By incorporating tailored GPEs, notable improvements have been achieved in various types of zinc batteries, including zinc–air batteries (ZABs), aqueous zinc-ion batteries (AZIBs), and fiber-shaped Zn-PANI batteries (Fs-ZPBs). GPEs have demonstrated the capability to enhance the mechanical stability, ionic conductivity, and cyclic stability of batteries, ultimately optimizing the overall performance of zinc batteries. Noteworthy achievements include the utilization of polyacrylamide (PAM)-based alkaline gel electrodes to improve power density in flexible ZABs, the development of polyvinyl alcohol–glycerol (PVA/G) GPEs for AZIBs with wide temperature stability, and the use of methanesulfonic acid (MSA) to improve the stability and ionic conductivity of PVA-based GPEs in Fs-ZPBs. Additionally, the introduction of multinetwork cross-linked composite GPEs and the incorporation of hollow Sn-inner HPβCD have demonstrated promising results in enhancing the performance and cyclic lifespan of zinc–air batteries. These advancements pave the way for the future development of efficient, safe, and flexible zinc-based energy storage systems.

## 8. Na-Ion Batteries

Sodium-ion batteries (SIBs) are a viable replacement for lithium-ion batteries (LIBs) due to the widespread availability of sodium and cheaper manufacturing costs. Gel polymer electrolytes (GPEs) with superior ionic conductivities at ambient temperatures, however, have been investigated due to safety concerns over the combustibility of organic liquid electrolytes that are comparable to LIBs. To advance the widespread commercialization of SIBs, significant efforts have been dedicated to developing GPE-based SIBs that offer high levels of ionic conductivity, mechanical stability, and cyclic stability [87]. In Table 6, a comprehensive analysis is provided, outlining the comparative aspects of various GPE compositions for Na-ion batteries, including the method of preparation, specific capacity, cyclic stability, and ionic conductivity.

To enhance mechanical stability and mitigate dendrite formation, a hydroxyapatite (HA)-incorporated polyvinylidene fluoride-co-hexafluoropropylene (PVDF-HFP) gel polymer electrolyte (GPE) was employed in sodium-ion batteries (SIBs). HA, an environmentally friendly biomaterial known for its mechanical stability and functional groups, offers favorable characteristics for composite structure formation, thereby improving the longevity and safety of SIBs. Specifically, a blend of HA-incorporated PVDF-HFP and poly(butylmethacrylate) (PBMA) was dissolved in a mixture of *N,N*′-dimethylformamide and acetone (3:7 ratio), and the GPE was formed via solution casting. This blend was obtained via solution blending. The obtained blend showed an ionic conductivity of 1.086 × 10^−3^ S cm^−1^. Carbon-coated sodium and sodium metal were employed as the cathode and anode, respectively. The fabricated SIB cell demonstrated a relatively high specific capacity of 109 mA h/g at 1C, attributed to its improved interfacial stability, excellent ionic stability, and low electrolyte leakage. The cyclic stability after 500 cycles was 71.7%. Furthermore, this blend GPE can serve as both a separator and electrolyte in SIBs [88].

**Figure 14:**
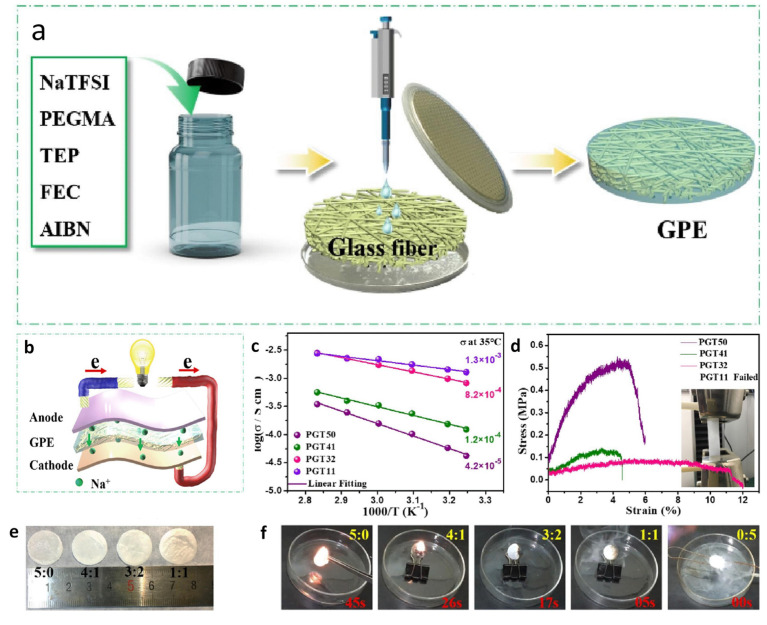
(**a**) A diagram depicting the preparation process of GPEs. (**b**) A schematic representation of flexible sodium batteries. (**c**) The modification of ionic conductive properties in GPEs with temperature. (**d**) Stress−strain curves showing the mechanical behavior of GPEs. (**e**) Photographic images showcasing the appearance of GPEs. (**f**) Flammability experiments conducted on GPEs and a TEP solution. Reproduced with permission from [89].

Gel polymer electrolytes (GPEs) have proven to be highly effective in enhancing the ionic conductivity of sodium-ion batteries (SIBs), surpassing values of 10^−4^ S/cm. In earlier methods, the polymer matrix was plasticized using carbonate solvents such as ionic liquids, propylene carbonate, ethylene carbonate, and dimethyl carbonate. However, these solvents were flammable and not suitable for large-scale applications. To address this, organic phosphate-based solvents have emerged as a promising alternative due to their nonflammable nature, high solvating capability, wide electrochemical window, low cost, and viscosity. In a study by Chen et al., triethyl phosphate (TEP) was utilized as a flame-resistant solvent for the preparation of a poly(ethylene glycol) methyl ether methacrylate (PEGMA) based GPE for SIBs. Figure 14a,e,f depict a schematic illustration of the preparation process, digital photographs, and flammability tests of GPEs. 

In the fabrication of the GPE, PEGMA was mixed with sodium bis(fluorosulfonyl)imide (NaFSI), TEP, a film-forming additive (fluoroethylene carbonate), and 2,2-azoisobutyronitrile. The mixture was then subjected to thermal-induced polymerization at 80 °C for at least 3 h. Figure 14c,d show the stress–strain profiles of GPEs and the temperature dependences of their ionic conductivities. The optimized GPE composition with a 3:1 PEGMA to TEP ratio and a film-forming agent volume of 5% demonstrated a high ionic conductivity of 0.91 mS/cm. Additionally, a wide electrochemical window of 4.8 V was achieved. SIB cells assembled with this GPE exhibited a discharge capacity of 102 mA h/g at 0.2 C and maintained a capacity retention of 91% after 400 cycles. The maximum observed ionic conductivity was 9.1 × 10^−4^ S/cm at 27 °C, which closely approaches the ionic conductivity values of liquid electrolytes (ranging from 10^−3^ to 10^−2^ S/cm) [89].

Due to their high degree of safety, favorable transport characteristics, excellent electrochemical stability, and low volatility, sodium-ion batteries (NIBs) have gained significant attention in recent years. Shadma Parveen et al. produced a gel polymer electrolyte (GPE), using solution casting and a mixture of the sodium salt of TFSI (NaTFSI), diglyme (G2), and poly(vinylidene fluoride-co-hexafluoropropylene) (P(VDF-HFP)) dissolved in acetone. The performance of this sodium-ion conducting GPE film was evaluated in NIB cells, with commercial Na_0.7_CoO_2_ serving as the cathode and Na metal foil as the anode. The GPE exhibited an electrochemical stability window of approximately 5.2 V, a high ionic conductivity of around 1.12 × 10^−3^ S/cm at room temperature, a large Na transference number of approximately 0.58, and thermal stability up to approximately 100 °C. The initial discharge capacity of the NIB cell was measured to be 91.76 mA h/g at a rate of 0.05 C. Over 40 cycles at 0.05 C, the Na metal anode displayed reversible discharge, with a capacity of around 60 mA h/g. Furthermore, extraction or deposition tests indicated excellent stability, with a polarization value limit of ±19 mV. The extraction of diglyme’s maximum occupied molecular orbital (HOMO) electrons by the Na salt was predicted to increase the oxidation stability of the metal–diglyme complexes. This suggests that the insertion of NaTFSI into the diglyme contributed to the improved oxidation stability observed in the system [90].

The use of gel polymer electrolytes (GPEs) in sodium-ion batteries (SIBs) has demonstrated remarkable advancements in improving their mechanical stability, ionic conductivity, and cycling performance. The incorporation of hydroxyapatite (HA) in a polyvinylidene fluoride-co-hexafluoropropylene (PVDF-HFP) GPE demonstrated enhanced interfacial stability and reduced electrolyte leakage in SIBs. The resulting composite structure, formed through a solution casting process, exhibited a favorable ionic conductivity and facilitated the use of the blend GPE as both a separator and electrolyte. Similarly, the utilization of organic phosphate-based solvents, such as triethyl phosphate (TEP), in a poly(ethylene glycol) methyl ether methacrylate (PEGMA) showcased a nonflammable alternative with a high solvating capability and wide electrochemical window. The optimized composition demonstrated impressive ionic conductivity and enabled the SIB cells to maintain good retention even after repeated cycles. Furthermore, the development of a sodium-ion-conducting GPE using poly(vinylidene fluoride-co-hexafluoropropylene) (PVDF-HFP) and the sodium salt of TFSI (NaTFSI) exhibited a high ionic conductivity, excellent electrochemical stability, and thermal stability. The NIB cells incorporating this GPE demonstrated promising initial discharge capacity and reversible discharge with remarkable stability. These results demonstrate the potential of GPEs in advancing the performance and safety of sodium-ion batteries, bringing us closer to practical and efficient energy storage solutions for various applications. Further research and optimization of GPE formulations and design are crucial for realizing the full potential of sodium-ion batteries and expanding their practical applications in the future.

## 9. Dual-Ion, Solar and Organic Batteries

GPEs (gel polymer electrolytes) have been extensively explored, not only in lithium-ion batteries but also in other emerging battery technologies such as dual-ion batteries, solar batteries, and all-organic batteries. A dual-ion battery, also known as a double-ion battery or symmetric battery, is a type of energy storage system in which both cations and anions are involved in charge transfer during battery operation. Unlike conventional batteries, in which only one type of charge carrier (either cations or anions) is responsible for the electrochemical reactions, dual-ion batteries utilize both types of charge carriers to enhance their overall efficiency and energy density. In a dual-ion battery, the electrolyte plays a crucial role in providing the necessary charge carriers for the electrochemical reactions. During the charging and discharging processes, the quantities of both anions and cations in the electrolyte might change. Therefore, the development of suitable GPEs becomes essential to facilitate the efficient transport of both cations and anions in these systems. GPEs offer several advantages in dual-ion batteries. Firstly, they can provide a stable and homogeneous environment for ion transport, allowing for improved ionic conductivity and reduced interfacial resistance. Secondly, GPEs can help mitigate the diffusion mismatch between different ions, ensuring balanced ion transport and preventing ion depletion at the electrode–electrolyte interfaces. Thirdly, GPEs can contribute to enhanced cycling stability and improved safety by inhibiting the development of dendrites, which results in battery failure and short circuits. Moreover, GPEs have been explored in solar batteries and all-organic batteries, which are promising alternatives to traditional inorganic-based energy storage systems. In solar batteries, GPEs can serve as solid-state electrolytes that enable efficient charge separation and transport within the device. In all-organic batteries, GPEs based on organic polymers offer advantages such as high flexibility, tunable properties, and compatibility with organic electrode materials [75]. Table 7 outlines the comparative aspects of various GPE compositions, including the method of preparation, specific capacity, cyclic stability, and ionic conductivity for diverse battery systems.

In dual-ion batteries (DIBs), the intercalation of anions has been found to be detrimental to the structure of the graphite electrode. This phenomenon leads to early capacity decay, short cyclic stability, and volume expansion issues. To address the challenge of volume expansion, Wang et al. developed a microporous gel polymer electrolyte (GPE) based on polyvinylidene fluoride-hexafluoropropylene (PVDF-HFP) for solid-state DIBs. In their study, a microporous PVDF-HFP film was synthesized using the phase inversion method. The PVDF-HFP solution in N-methyl-2-pyrrolidinone was mixed with silicon dioxide (SiO_2_) particles and a pore-forming agent (dibutyl phthalate). The resulting solution was cast onto an aluminum (Al) foil substrate and subjected to phase inversion via the introduction of methanol. The resulting film was then immersed in a liquid electrolyte containing lithium hexafluorophosphate (LiPF_6_). The microporous GPE exhibited high levels of ionic conductivity and mechanical strength, providing sufficient ions for efficient charge transport and enabling better contact with the Al foil anodes. The GPE also played a crucial role in reducing gas generation and protecting the Al foil from corrosion. Remarkably, the dual-ion battery using the microporous GPE demonstrated excellent cycling stability, with 1000 cycles retaining 82% of the original capacity (80 mA h/g). Additionally, the dual-ion battery showed an elevated working voltage of 4.2 V, expanding its potential applications. The maximum ionic conductivity observed for the PVDF-HFP-based GPE with a SiO_2_ content of 15% at room temperature was measured to be 2.40 mS/cm with LiPF_6_ as the electrolyte. These remarkable results emphasize the potentiality of the dual-ion cell with a microporous GPE for future eco-friendly energy storage applications [91].

To replace liquid electrolytes in rechargeable quasi-solid-state dye-sensitized solar batteries, Bao Lei et al. employed a poly(ethylene oxide) (PEO) gel polymer electrolyte (GPE). This type of battery is capable of converting solar energy into chemical energy when exposed to light. The GPE was prepared by dissolving PEO and LiClO_4_ in a solvent mixture comprising ethylene carbonate and propylene carbonate, which served as the anode electrolyte. The cathode electrolyte was prepared by incorporating the required amount of 4-tert-butylpyridine into the aforementioned GPE. In dark conditions, when a constant current discharge occurred, the chemical energy that was stored in this battery was transformed back into electrical energy. The inclusion of PEO GPE in the system improved its stability. Specifically, the GPE formulation containing 7% PEO exhibited the highest ionic conductivity, measuring 3.88 × 10^−4^ S cm^−1^. Comparatively, when using liquid electrolytes, the capacity retention after 19 cycles was only 52.9%. In contrast, with the implementation of a PEO GPE, even after 30 cycles, 86.3% of the starting capacity remained [92].

In pursuit of developing printable gel polymer electrolytes (GPE) for all-organic batteries, Muench et al. successfully formulated a versatile solution. Addressing challenges related to solid electrolyte interface (SEI) formation, electrolyte stability, Li dendrite development, and Li salt requirements, their work introduced a promising approach. By harnessing the unique characteristics of ionic liquids (ILs) as both solvents and salts, the complexity of the electrode system could be simplified. Furthermore, ILs offered additional benefits, including high levels of ionic conductivity and thermal stability, and negligible vapor pressure, making them well-suited for all-organic electrolytes. To achieve their goal, the researchers developed a printable GPE ink formulation comprising nanofillers, methacrylate monomers, and a cross-linking agent. Specifically, benzyl methacrylate, poly(ethylene glycol) methylether methacrylate, triethylene glycol dimethacrylate, and 1-butyl-3-methylimidazolium bis(trifluoromethylsulfonyl)imide (BMImTFSI) were blended and placed between two siliconized PET foils. By adjusting the spacer for the desired film thickness, the system underwent photopolymerization under UV light for a period of 10 to 60 min. This allowed the formation of a mechanically stable, flexible GPE with an ionic conductivity of around 0.74 S/cm. Remarkably, the printed GPE served a dual purpose as both the electrolyte and the separator in all-organic batteries. It exhibited a maximum specific discharge capacity of 24 mA h/g (at 0.1C), with 77% of the initial capacity retained after 1000 cycles. The mechanical stability of the GPE, as demonstrated through an indentation analysis, enabled its reliable function as a separator, even during the polymerization process in the presence of oxygen. Overall, this innovative printable GPE formulation presents a promising pathway for the advancement of all-organic batteries [93].

The development of gel polymer electrolytes (GPEs) has shown great potential in advancing the performance and endurance of diverse types of batteries. In the context of dual-ion batteries (DIBs), the utilization of a microporous GPE based on polyvinylidene fluoride-hexafluoropropylene (PVDF-HFP) has addressed challenges relating to volume expansion and electrode structure deterioration. The microporous GPE exhibited high levels of ionic conductivity and mechanical strength with improved contact with the anode, which it protected from corrosion, resulting in an excellent cycling stability and a high working voltage. Similarly, in quasi-solid-state dye-sensitized solar batteries, the incorporation of a poly(ethylene oxide) (PEO) GPE enhanced the durability and ionic conductivity of the system, leading to significantly enhanced capacity retention and cycling performance compared to liquid electrolytes. Furthermore, in the pursuit of printable GPEs for all-organic batteries, researchers successfully formulated a versatile solution using ionic liquids (ILs) as solvents and salts. The resultant printed GPE exhibited impressive mechanical stability, ionic conductivity, and specific discharge capacity, serving as both the electrolyte and separator in the all-organic batteries. These advancements in GPE technology offer promising opportunities for the advancement of eco-friendly energy storage technologies, providing improved performance and reliability.

## 10. Conclusions and Future Scope

The development of gel polymer electrolytes (GPEs) has demonstrated significant potential in enhancing the performance, stability, and safety of various types of batteries. The advancements in GPE technology have addressed key challenges in lithium-ion batteries (LiBs), lithium metal batteries (LMBs), lithium–oxygen batteries, lithium–sulfur batteries, zinc-based batteries, sodium-ion batteries, and dual-ion batteries. Through the utilization of innovative strategies such as alternative precursors, 3D printing techniques, doping, composite designs, the incorporation of ionic liquids, and the introduction of renewable and biodegradable materials, researchers have achieved notable improvements in multiple aspects of battery performance. In LiBs and LMBs, GPEs have resulted in stable Li-electrolyte interfaces, dendrite-free morphologies, and enhanced stripping/plating efficiencies and improved the interfacial chemistry and cycling performance. Additionally, the incorporation of organic electrolytes, coiling GPE membranes, fireproof gel electrolytes, and biodegradable nanofiber membranes has addressed interfacial issues, enhanced anode stability, and facilitated continuous Li ion transmission.

For lithium–oxygen batteries, the development of hybrid solid–gel electrolytes and GPEs has shown promise in improving cyclic stability, interfacial stability, and specific capacity. The use of lithiophilic modifications and super-high ionic conductive gel polymers (SHGPs) has addressed challenges relating to lithium dendrite growth, lithium–sulfur batteries, and interface interactions. In zinc-based batteries, GPEs have enhanced mechanical stability, ionic conductivity, and cyclic stability. Notable achievements include improvements in zinc–air batteries, aqueous zinc-ion batteries, fiber-shaped Zn-PANI batteries, and the utilization of multinetwork cross-linked composite GPEs. In sodium-ion batteries, GPEs have shown advancements in mechanical stability, ionic conductivity, and cycling performance. The incorporation of hydroxyapatite, organic phosphate-based solvents, and the sodium salt of TFSI has improved interfacial stability, solvating capability, and thermal stability. Furthermore, in dual-ion batteries, microporous GPEs and poly(ethylene oxide) GPEs have addressed challenges relating to volume expansion, electrode structure deterioration, and stability in quasi-solid-state dye-sensitized solar batteries. The utilization of ionic liquids as solvents and salts has enabled the development of printable GPEs for all-organic batteries demonstrating impressive mechanical stability, ionic conductivity, and specific discharge capacity. These advancements collectively contribute to the development of high-performance, safe, and eco-friendly energy storage systems. 

Looking ahead, the future scope of gel polymer electrolytes lies in the continued exploration and optimization of new materials, fabrication techniques, and architectures to further enhance the electrochemical performance, mechanical stability, and safety of GPEs. The development of advanced characterization techniques will enable a better understanding of the ion transport mechanisms, interfacial interactions, and degradation mechanisms within GPE-based battery systems. The incorporation of nanofillers, such as graphene, carbon nanotubes, and metal oxides, can improve the ionic conductivity, mechanical strength, and stability of GPEs. Additionally, the utilization of advanced additives, such as functionalized polymers, ionic liquids, and nanoscale solid electrolyte interphases (SEIs), can further optimize the performance and safety of batteries utilizing GPEs. Scalable and cost-effective manufacturing processes, such as roll-to-roll processing and inkjet printing, are crucial for the practical adoption of GPEs in large-scale energy storage systems. These advancements will contribute to the commercial viability of GPE-based batteries and accelerate their integration into electric vehicles, portable electronics, and grid energy storage. With ongoing research and development, GPEs have prospects for enabling the next generation of high-energy-density, long-lasting, and safe energy storage systems. The future of GPEs lies in continued material optimization, advanced manufacturing techniques, and their integration into various applications, driving the transition towards a more sustainable and electrified future.

## Figures and Tables

**Table 1:** Comparison of different GPE compositions, their methods of preparation, specific capacities, cyclic stabilities, and ionic conductivities for Li-ion batteries.

S. No.	GPE Composition	Method of Preparation	Specific Capacity (mA h/g)	Cyclic Stability	Ionic Conductivity (S/cm)	Ref. (Publication Year)
1	PVDF-HFP/LiSnOS	Solution casting	134.6 at 0.2 C	Stable for 30 cycles	1.92 × 10^−4^	[52] (2019)
2	Li-TFSI/EMI-TFSI/P(HEMA)	Photopolymerization	100 mA h/g (0.1 C)	Up to 15 cycles (at 0.1 C)	2.9 × 10^−3^ at 25 °C	[53] (2021)
3	PEGDA/Ta doped Li_7_La_3_Zr_2_O_12_	In situ polymerization	104.1 mA h/g (at 0.5 C)	82.6% after 100 cycles	2.37 × 10^−3^ at R.T.	[54] (2022)
4	PEO/PU/LiTFSI	Solution casting	170 mA h/g at 0.1 C, at 60 °C	96.1% after 100 cycles	1.33 × 10^−3^ at 60 °C	[55] (2022)
5	PDOL/LiTFSI/LiPF_6_	In situ polymerization	153.7 mA h/g (at 1 C)	Up to 500 cycles	~0.6 × 10^−2^ at R.T.	[56] (2023)
6	PVDF-HFP/HMSFs	Phase inversion	2590.8 mA h/g (at 0.3 C)	50% after 500 cycles	1.2 × 10^−3^ at R.T.	[57] (2023)

**Table 2:** Comparison of different GPE compositions, their methods of preparation, specific capacities, cyclic stabilities, and ionic conductivities for Li-metal batteries.

S. No.	GPE Composition	Method of Preparation	Specific Capacity (mA h/g)	Cyclic Stability	Ionic Conductivity (S/cm)	Ref. (Publication Year)
1	PEO/PA/TEGDME/LiTFSI	Photopolymerization	140 mA h/g (at 0.5 C)	88.57% after 100 Cycles	6.4 × 10^−4^ at 25 °C	[59] (2018)
2	PVDF-HFP/PP13TFSI/LiTFSI	Solution casting	128.7 mA h/g	Up to 400 cycles	-	[60] (2020)
3	PVDF-HFP/PEA/PP13TFSI	Photopolymerization	153.7 mA h/g (0.5 C)	92.7% after 500 cycles	9.5 × 10^−4^ at R.T	[61] (2021)
4	PVDFHFP)/LiTFSI	Solution casting	87.5 mA h/g (at 0.5 mA/cm)	93.26% after 830 cycles	3.11 ×10^−5^ at R.T	[62] (2021)
5	Mxenes/PVDF-HFP/LiTFSI	Solution casting	900 mA h g^−1^ (at 0.5 C)	74% after 500 cycles	8.1 × 10^−4^	[63] (2022)
6	PDOL/LiTFSI in PAN/PLLA matrix	Electrospinning/Immersion	151.79 mA h/g (at 1 C)	98.19% after 100 cycles	1.2 × 10^−3^	[64] (2022)
7	E2BADMA/PEGDA/LiTFSI	In situ polymerization	170 mA h/g (at 0.1 C)	82.35% after 100 cycles).	4.4 × 10^−4^	[65] (2022)
8	PVDF-HFP)/CA/SN/ZIF-8 NPS	Electrospinning/Solution casting	257.5 (at 0.2 C)	84.6% after 500 cycles	3.44 × 10^−3^	[66] (2022)
9	PEGDMA/TFMA/LIF	In situ thermal polymerization	170 (at 0.5 C)	90% after 1000 cycles	3.1 × 10^−3^	[67] (2022)
10	P(VDF-HFP), PEGDGE, and EMITFSI	In situ polymerization	162 (at 0.1 C, 60 °C)	96.1% after 50 cycles (at 60 °C)	1.69 × 10^−3^ at R.T	[68] (2022)
11	PEGMEA/LiTFSI/Liquid carbonates	In situ polymerization	158.5 (at 0.5 C).	98.87% after 100 cycles	1.73 × 10^−4^ at 60 °C	[69] (2022)
12	PVDF-HFP/LiTFSI	Solution casting	870.3 (at 0.1 C)	48.28% after 20 cycles	8.0 × 10^−4^ at 25 °C	[70] (2022)
13	PETEA/Cystine/ICEM/LiTFSI	In situ polymerization	135.4 (at 0.1 C)	Stability > 1200 h	1.1 × 10^−3^ at 25 °C	[71] (2022)

**Table 3:** Comparison of different GPE compositions, their methods of preparation, specific capacities, cyclic stabilities, and ionic conductivities for Li-oxygen batteries.

S. No.	GPE Composition	Method of Preparation	Specific Capacity (mA h/g)	Cyclic Stability	Ionic Conductivity (S/cm)	Ref. (Publication Year)
2	P(VDF-HFP)/LiTFSI	Solution casting	-	-	9.44 × 10^−5^	[35] (2021)
3	PI/LiTFSI	Phase Inversion	6.39 mA h/cm^2^	Up to 366 cycles	4.4 × 10^−4^	[74] (2023)
4	CA-LiTFSI	Phase Inversion	6.98 mA h/cm^2^ at 0.1 mA/cm^2^	Up to 370 cycles	4.7 × 10^−4^	[75] (2022)

**Table 4:** Comparison of different GPE compositions, their methods of preparation, specific capacities, cyclic stabilities, and ionic conductivities for Li-sulfur batteries.

S. No.	GPE Composition	Method of Preparation	Specific Capacity (mA h/g)	Cyclic Stability	Ionic Conductivity (S/cm)	Ref. (Publication Year)
1	PVDF/PDA/LiTFSI	Phase inversion	1215.4 (at 0.1 C)	71.48% after 200 Cycles	5.71 × 10^−4^ at R.T.	[78] (2018)
2	PEDGE/PEI/LiTFSI	Solution casting	950 (at 0.2 C)	98% after 100 Cycles	0.75 × 10^−3^ at R.T.	[79] (2019)

**Table 5:** Comparison of different GPE compositions, their methods of preparation, specific capacities, cyclic stabilities, and ionic conductivities for zinc batteries.

S. No.	GPE Composition	Method of Preparation	Specific Capacity (mA h/g)	Cyclic Stability	Ionic Conductivity (S/cm)	Ref. (Publication Year)
1	PAM-AGE	Solution casting	720	-	215.6 × 10^−3^ S/cm^2^	[82] (2020)
2	PVA/Glycerol	Solution casting	406.4 (at 0.2 C)	96% after 300 cycles	18.2 × 10^−3^ at 25 °C	[83] (2020)
3	PVA/MSA/PANI	Solution casting	100.3 (at 2 C)	88.1% after 2000 cycles	-	[84] (2021)
4	PAA/PVA/Cellulose	Solution casting	724 (at 3 mA/cm^2^)	Up to 56 h	0.123	[85] (2023)

**Table 6:** Comparison of different GPE compositions, their methods of preparation, specific capacities, cyclic stabilities, and ionic conductivities for Na-ion batteries.

S. No.	GPE Composition	Method of Preparation	Specific Capacity (mA h/g)	Cyclic Stability	Ionic Conductivity (S/cm)	Ref. (Publication Year)
1	PVDF-HFP/PBMA/HA	Solution casting	109 mA h/g (at 1 C)	71.7% after 500 cycles	1.086 × 10^−3^	[88] (2020)
2	PEGMA/NaFSI	Thermal induced polymerization	102 mA h/g (at 0.2 C)	91% after 400 cycles	9.1 × 10^−4^ at 27 °C	[89] (2020)
3	P(VDF-HFP)/G2/NaTFSI	Solution casting	91.76 mA h/g (at 0.05 C)	65.39% after 40 cycles	1.12 × 10^−3^ at R.T.	[90] (2022)

**Table 7:** Comparison of different GPE compositions, highlighting the methods of preparation, specific capacities, cyclic stabilities, and ionic conductivities for different battery systems.

S. No.	GPE Composition	Method of Preparation	Specific Capacity (mA h/g)	Cyclic Stability	Ionic Conductivity (S/cm)	Type of Battery	Ref. (Publication Year)
1	PVDF-HFP)/LiPF_6_	Phase inversion	80	82% after 1000 Cycles	~2.40 mS/cm at R.T.	Dual-ion	[91] (2018)
2	PEO/LiClO_4_	Solution casting	8	86.3 after 30 Cycles	3.88 × 10^−4^ S/cm	Solar battery	[92] (2019)
3	Methacrylate polymer/BMImTFSI	In situ polymerization	24 (at 0.1 C)	77% after 1000 cycles	0.74 S/cm at R.T.	Organic battery	[93] (2020)

## Data Availability

Not applicable.
